# Metagenomic Insights Into the Taxonomic and Functional Features of *Kinema*, a Traditional Fermented Soybean Product of Sikkim Himalaya

**DOI:** 10.3389/fmicb.2019.01744

**Published:** 2019-08-02

**Authors:** Jitesh Kumar, Nitish Sharma, Girija Kaushal, Sanjukta Samurailatpam, Dinabandhu Sahoo, Amit K. Rai, Sudhir P. Singh

**Affiliations:** ^1^Center of Innovative and Applied Bioprocessing, Mohali, India; ^2^Institute of Bioresources and Sustainable Development, Sikkim Centre, Tadong, India; ^3^Institute of Bioresources and Sustainable Development, Imphal, India

**Keywords:** *Kinema*, metagenome, soybean, fermented food, functional food, taxonomic profiling, functional potential

## Abstract

*Kinema* is an ethnic, naturally fermented soybean product consumed in the Sikkim Himalayan region of India. In the present study, the whole metagenome sequencing approach was adopted to examine the microbial diversity and related functional potential of *Kinema*, consumed in different seasons. Firmicutes was the abundant phylum in *Kinema*, ranging from 82.31 to 93.99% in different seasons, followed by Actinobacteria and Proteobacteria. At the species level, the prevalent microorganisms were *Bacillus subtilis*, *Bacillus amyloliquefaciens*, *Bacillus licheniformis*, *Corynebacterium glutamicum*, *Bacillus pumilus*, and *Lactococcus lactis*. The abundance of microbial species varied significantly in different seasons. Further, the genomic presence of some undesirable microbes like *Bacillus cereus, Proteus mirabilis*, *Staphylococcus aureus*, *Proteus penneri, Enterococcus faecalis*, and *Staphylococcus saprophyticus*, were also detected in the specific season. The metagenomic analysis also revealed the existence of bacteriophages belonging to the family *Siphoviridae*, *Myoviridae*, and *Podoviridae*. Examination of the metabolic potential of the *Kinema* metagenome depicted information about the biocatalysts, presumably involved in the transformation of protein and carbohydrate polymers into bioactive molecules of health-beneficial effects. The genomic resource of several desirable enzymes was identified, such as β-galactosidase, β-glucosidase, β-xylosidase, and glutamate decarboxylase, etc. The catalytic function of a novel glutamate decarboxylase gene was validated for the biosynthesis of γ-aminobutyric acid (GABA). The results of the present study highlight the microbial and genomic resources associated with *Kinema*, and its importance in functional food industry.

## Introduction

Fermented soybean products are widely consumed in many Asian countries due to their flavor, aroma, and health promoting properties (Sourabh et al., 2015). Natural fermentation is carried out in diverse traditional ways, resulting in a variety of products such as *natto*, *miso* (Japan), *cheonggukjang*, *doenjang, kanjang*, *meju* (Korea), *tempeh* (Indonesia), *douchi*, *sufu*, *doubanjiang* (China), *thua*-nao (Thailand), *Kinema*, *hawaijar*, and *tungrymbai* (India) (Jeyaram et al., 2008; Rai and Jeyaram, 2015). *Kinema* is prepared through spontaneous fermentation of cooked yellow soybean seeds (*Glycine max*) in the Sikkim Himalayan region of India. Fermentation is promoted by the diverse group of microorganisms present in the natural environment and raw material used for *Kinema* fermentation (Sarkar et al., 1997, 2002; Jeyaram et al., 2008).

Fermented soybean products are of great interest due to the presence of various health-promoting bioactive compounds produced during microbial fermentation. The role of microbes isolated from the fermented soybean products have been elucidated in the production of various bioactive substances such as polyphenols (isoflavones, phenolic acids and flavanols), bioactive peptides, γ-amino butyric acid, antimicrobial compounds, fibrinolytic enzymes, vitamins and exopolysaccharides (Cho Y.S. et al., 2011; Sanjukta and Rai, 2016; Rai et al., 2017). Taxonomical profile of cultivable microbes from *Kinema* fermentation has revealed the dominance of *Bacillus subtilis*, *Bacillus licheniformis*, *Bacillus circulans*, *Bacillus amyloliquefaciens*, *Bacillus thuringiensis*, *Bacillus cereus*, and *Bacillus sphaericus* (Sarkar et al., 2002; Tamang, 2015). Culture-independent techniques, based on 16S rRNA genes or whole metagenome sequencing, have been extensively used to understand the taxonomic profile of various fermented foods (Jung et al., 2011; Zhang et al., 2016; Wang and Shao, 2018). Whole metagenome sequencing provides an insight into the taxonomic profile and functional potential of the analyzed sample (Kaushal et al., 2018). It has been applied for the discovery of novel genes from the metagenome of diverse environmental samples (Thies et al., 2016; Danso et al., 2018; Kaushal et al., 2018). Similar applications of whole metagenome sequencing includes identification of the food-borne pathogen, exploration of the taxonomical and functional features of food microbiomes (Sulaiman et al., 2014; Rantsiou et al., 2018). Metagenomic exploration is limited to a few fermented foods, such as fermented tofu, whey (Fei et al., 2018), and kimchi (Jung et al., 2011). The microbiome community profile of *Kinema* has been previously studied using 16S rRNA sequencing (Sarkar et al., 2002). However, whole-metagenome based investigation, examining the taxonomic details and functional potential, of this fermented food product has not been done.

The sequencing-based metagenomic investigation gives the molecular insights about the effect of external factors that are crucial in determining microbial community dynamics, such as seasonal changes, and surrounding temperature (Jung et al., 2011; Lee et al., 2014). Sikkim Himalaya region faces diverse climatic conditions, ranging from cold weather (with minimum temperatures dipping to 4^∘^C), spring, and rainy seasons. We hypothesized that seasonal changes in Sikkim Himalaya region affect microbial community structure, and their functional potential during *Kinema* fermentation. The present study aims at investigating the taxonomic and functional profiling of the fermented soybean food product, *Kinema*, and exploring the genomic resource for microbial enzymes, useful in the biosynthesis of the desirable biomolecules of health-potential.

## Materials and Methods

### Sample Collection

*Kinema* samples (200–250 g) were collected from the local market of Lal Bazaar, Gangtok, Sikkim, India, for two consecutive years from December 2014 to October 2016. The sample collection was performed in four phases, i.e., during September to October (SO), December to January (DJ), March to April (MA), and June to July (JJ), keeping in mind the seasonal variation associated with these months. In each phase, six samples were collected at an interval of 1 week. These six sample fractions, belonging to one seasonal-phase, were pooled in equal quantity, and the mixture was treated as one sample for metagenomic investigation. The samples were placed in sterile bags, and stored at −80^∘^C until further use.

### Total DNA Extraction

Kinema samples were homogenized in Tris buffer (pH 8.0), taking the sample and buffer ratio of 1:20. The homogenate was filtered through four layers of sterile cheesecloth. The filtrate was then passed through 10 μm membrane filter (Merck Millipore Ltd., Germany) to remove any possible contamination of cells of plant origin. The filtered extracts were centrifuged, and the pellets were subjected to total DNA extraction using GeneJET Genomic DNA purification kit (Thermo Fisher Scientific, United States), following the manufacturer’s protocol. The quality and quantity of total DNA were assessed on 1% agarose gel electrophoresis and nanodrop (Thermo Fisher Scientific, United States).

### Metagenome Sequencing and *de novo* Assembly

Whole metagenome sequencing was done using 200 ng of total DNA. The paired end-sequencing library was prepared using the TruSeq Nano DNA Library Prep Kit (Illumina, United States). The library was prepared with 200 ng of total DNA. DNA was sheared in smaller fragments by using a Covaris instrument, followed by end-repair and 3′ A tail addition. The adaptor was ligated to the ends of the A-tailed DNA, and PCR amplification was performed using HiFi PCR Master Mix. The prepared library was sequenced on the Illumina Hiseq 2500 platform (2 × 150 bp chemistry) (Illumina, United States), following the standard instructions^1^. The quality score was calculated for the samples on a Phred scale in CLC genomics workbench (Qiagen, United States). The quality-filtered metagenomic read datasets were deposited in NCBI Sequence Read Archive (SRA) database with accession number: PRJNA529966.

*De novo* assembly of high-quality paired-end reads was performed after removing adaptor sequences using CLC genomics workbench 7.5.1 software (Qiagen, Valencia, CA, United States) at the default parameter. The assembly statistics were derived using the in-house Perl scripts. The ORFs were predicted from assembled scaffolds using Prodigal (v2.6.3) (Hyatt et al., 2010) in metagenome mode at default parameters.

### Taxonomical and Functional Profiling

The distribution of microbial community was identified by taking Prodigal derived open reading frames (ORFs) as input to Metagenomic Rapid Annotations using Subsystems Technology (MG-RAST) server^2^) (Meyer et al., 2008). The ORFs were annotated against the NCBI reference sequence (RefSeq) database in MG-RAST at the default parameters (i.e., *E*-value 1e^–5^ and identity 60%). The microbial communities were classified at different taxonomic levels- phylum, class, order, family, and genus. For functional categorization, the ORFs were mapped to KEGG Orthology (KO) and Clusters of Orthologous Group of Proteins (COGs) databases of MG-RAST pipeline at default parameters (i.e., *E*-value 1e^–5^ and identity 60%).

### Statistical Analysis

The species counts, and abundance of the KEGG functions were used for clustering of the samples following the principal component analysis (PCA). The PCA plot was visualized using ClustVIS web tool^3^. Correlation analysis was performed using the Spearman’s method in Microsoft excel using XLSTAT statistical add on package^4^.

### Estimation of Phage Abundance

The assembled scaffolds were used to identify putative bacteriophage sequences using Metaphinder tool^5^. The output files of Metaphinder were imported into Megan 6^6^, which uses the NCBI database for taxonomical classification. Megan 6 assigned viral taxa at the default parameters. Enrichment of the phages was computed using core biome feature in Megan 6.

### Gene Function Annotation

The metagenomic data was screened for the identification of novel putative genes by performing BLASTx analysis (*E*-value cutoff 1e^–5^) of the metagenomic contigs against different public protein databases, e.g., Carbohydrate Active Enzymes (CAZy)^7^ (Lombard et al., 2014), NCBI^8^, GO FEAT^9^, NCBI reference sequence (RefSeq)^10^, and MEROPS peptidase database^11^.

### Validation Experiments

The metagenome assembly was validated by performing polymerase chain reaction (PCR), using 50 ng metagenomic DNA as the template, and the gene-specific primers designed from the assembled metagenome data. The Q5^®^ High-Fidelity DNA Polymerase (NEB, United States) was used in the PCR reactions. The cycling conditions were 98^∘^C for 2 min, 30 cycles of 98^∘^C for 20 s, 60^∘^C for 30 s, 72^∘^C for 1:30 min, and the final extension at 72^∘^C for 5 min.

The PCR amplicon representing a putative gene encoding glutamic acid decarboxylase (GAD) was cloned in pJET1.2 vector (Thermo Fisher Scientific, United States), and sequenced. The sequence confirmed *GAD* insert was sub-cloned in the bacterial expression vector, pET28a, between the restriction sites, *Nde*I and *Xho*I. The gene-construct (pET28a-GAD) was transferred in *Escherichia coli* BL21 DE3 cells. The recombinant *E. coli* cell culture (∼0.5 O.D_600_) was induced for protein expression by introducing 0.1 mM Isopropyl β-D-1-thiogalactopyranoside (IPTG) in the culture broth. The induced cell culture was incubated at 16^∘^C and 150 RPM shaking (Eppendorf, United States) for 16 h, as described previously (Patel et al., 2016). The culture was centrifuged at 5000 RPM to obtain the cell-pellet. The cell-pellet was re-suspended in 20 mM Tris−HCl (pH 8.0) and 100 mM NaCl. Cell-lysis was performed in a probe-sonicator (Qsonica, United States), as mentioned previously (Patel et al., 2016). The crude extract was centrifuged at 10,000 RPM to sediment the cell debris. The cell-free crude extract was used to perform the catalytic reaction in 50 mM sodium acetate buffer (pH 6.0), containing 20 mM monosodium glutamate (MSG) (Himedia Lab, India) and 0.2 mM Pyridoxal 5′-phosphate (Himedia Lab, India), at 37^∘^C for 30 min. The reaction product and the standard were spotted on thin layer chromatography (TLC) plate (Merck Millipore, Germany). The TLC was run in a solvent system, comprised of n-butanol: acetic acid: water (4:1:1). The TLC plate was air-dried, and a solution of 0.2% ninhydrin prepared in 100% ethanol was evenly sprayed on the plate (Cho S. Y. et al., 2011). The TLC plate was incubated on a hot-plate until the appearance of the spots for the reaction product and the standards.

## Results

### Metagenome Sequencing and Assembly Statistics

The total DNA extracted from *Kinema* samples collected during different seasons was subjected to Illumina sequencing. The average Phred score of sequencing reads for all the four samples was calculated to be ≥30. A total of ∼8.5 to 24.5 million high-quality paired-end reads were generated, with an average read length of 150 bp, accounting for the data size of 1.2 to 3.6 Gb (Table 1). The high-quality reads were assembled into 31.8 to 36.6 thousand scaffolds. A total of 64.3 to 97.2 thousand ORFs were identified in the scaffolds (Table 1). The results of the sequencing and assembly statistics are summarized in Table 1.

**TABLE 1:** Summary of illumina sequencing and assembly statistics.

	**SO**	**DJ**	**MA**	**JJ**
Data size (Gb)	1.8	3.6	1.7	1.2
Number of high quality paired-end reads	12,027,810	24,503,206	11,378,074	8,554,254
Total number of bases	1,804,171,500	3,675,480,900	1,70,67,11,100	1,28,31,38,100
Number of scaffold	36,603	35,497	31,855	34,917
Total scaffold length (bp)	4,62,97,666	7,16,34,068	4,40,19123	3,75,00767
Average scaffold size (bp)	1265	2018	13812	1074
Scaffold N50 (bp)	2695	5094	5867	1851
Largest scaffold size (bp)	482,635	686,595	412,016	232,823
Minimum scaffold size (bp)	200	400	200	300
Number of ORFs (Prodigal)	73,276	97,267	68,344	64,383
Total mapped ORFs to MG-Rast	65,626	86,210	59,579	53,437
Average ORF length	555	638	563	509
Number of ORFs mapped to COG	31,421	36,899	26,674	20,656
Number of mapped ORFs mapped to KO	21,102	28,712	19,739	13,958
Number of predicted gene (NR)	66,599	89,497	61,185	54,216

### Taxonomic Profiling

**FIGURE 1:**
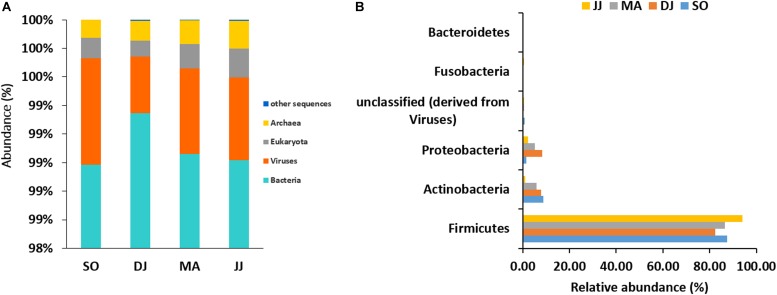
Taxonomic abundance of microbial community at the rank of domain **(A)**, and phyla **(B)** level. Top six abundant phyla are shown for representation.

The mapping of metagenomic ORFs against the RefSeq database of MG-RAST led to the identification of 48 phyla, 349 families, 746 genera, and 1685 species in the *Kinema* samples. A comparison of taxonomical profile at domain level revealed abundance of bacterial population, which ranged from 98.98 to 99.35%, followed by viruses (0.39 to 0.75%), eukaryota (0.11 to 0.2%), and archaea (0.12 to 0.19%) in *Kinema* samples from different seasons (Figure 1A and Supplementary Table S1). Among bacteria, Firmicutes (82.31 to 93.99%) was the abundant phylum, followed by Actinobacteria (1.05 to 8.87%) and Proteobacteria (1.55 to 8.22%) (Figure 1B and Supplementary Table S1). At the family level, the dominance of Bacillaceae (19.63 to 46.46%), Staphylococcaceae (4.10 to 26.77%) and Enterococcaceae (14.05 to 28.51%) was noticed (Supplementary Figure S1 and Supplementary Table S1). The taxonomic annotation revealed the predominance of *Bacillus*, ranging from 17.79 to 39.78%, followed by *Staphylococcus* (3.72 to 25.53%), and *Enterococcus* (14.05 to 28.51%) genera in the *Kinema* samples (Supplementary Figure S2 and Supplementary Table S1). *Bacillus* sp. is the key player of *Kinema* fermentation, and therefore, the abundance of its different species was evaluated. In the seasonal *Kinema* samples, *B. subtilis*, ranged from 6.65 to 12.02%, followed by *B. amyloliquefaciens* (1.04 to 6.95%), *B. licheniformis* (1.37 to 7.33%), and *B. cereus* (0.8–13.23%). The relatively higher representation of *B. cereus* (13.23%), and *B. subtilis* (12.02%) was observed in DJ, and SO samples, respectively. Other than *Bacillus* sp., the dominant species in *Kinema* included *Enterococcus faecalis* (5.10 to 11.25%), *Staphylococcus saprophyticus* (0.60 to 8.73%), *E. casseliflavus* (3.86 to 7.90%), and *E. faecium* (3.31 to 6.60%) (Supplementary Figure S3 and Supplementary Table S1). The other important species distinguished to contribute the *Kinema* microbiome were *Staphylococcus epidermidis* (0.67 to 3.98%), *Staphylococcus aureus* (0.74 to 3.83%), *E. gallinarum* (0.88 to 3.59%), *C. ammoniagenes* (0.02 to 3.20%)*, C. glutamicum* (0.04 to 1.11%), *B. pumilus* (0.51 to 0.93%), and *L. lactis* (0.52 to 0.96%). The ORFs representing *Corynebacterium* (0.26 to 7.77%), *Lactobacillus* (1.87 to 3.63%), *Clostridium* (1.78 to 3.19%), and *Streptococcus* (1.78 to 3.36%) were fewer in the metagenome.

**FIGURE 2:**
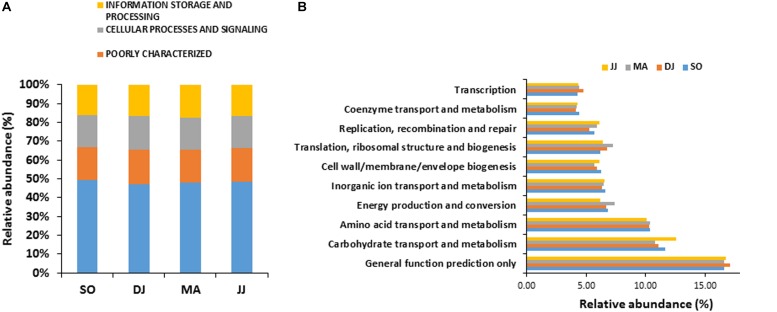
COG based mapping of metagenomics derived ORFs at level 1**(A)**, and level 2**(B)**. Top ten abundant phyla are shown for representation.

### PCA and Correlation Analysis

**FIGURE 3:**
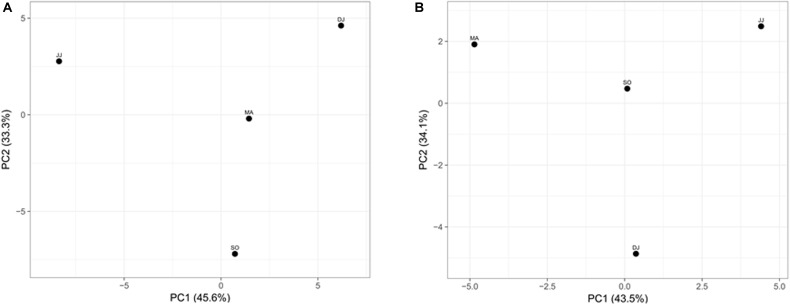
Principal component analysis of *Kinema* metagenomes based on microbial species abundance **(A)**, and KEGG functions **(B)**. The percentages of variance are represented at each axis.

Principal component analysis (PCA) was performed to examine the differential microbial species abundance in *Kinema* samples. Principal components were calculated for all the four seasonal *Kinema* samples. In the PCA plot, *Kinema* samples of different seasons were clustered separately (Figure 3A). The principal components, PC1 and PC2, showed a higher cumulative variance of 78.9%. The PCA plot analysis and high variance indicated a statistically significant difference in the profile of microbial community abundance among the seasonal *Kinema* samples.

A correlation was inferred among the taxa (at phylum and species levels) showing an abundance of 0.1% or above in the *Kinema* metagenomes (Supplementary Table S5). Spearman’s correlation analysis at phylum level revealed a positive correlation between Firmicutes and Crenarchaeota. Fusobacteria showed a positive correlation with Spirochaetes, Tenericutes, Synergistetes, Deferribacteres, and Lentisphaerae. Contrary to this, a negative correlation was observed among Actinobacteria, Bacteroidetes, Euryarchaeota, Cyanobacteria, Thermotogae, and Streptophyta.

At the species level, a positive correlation was inferred among *B. subtilis*, *Staphylococcus haemolyticus*, *Staphylococcus carnosus*, and *Exiguobacterium* sp. AT1b (Supplementary Table S5). *B. amyloliquefaciens* was found to be positively correlated with *Staphylococcus warneri*, *Staphylococcus lugdunensis*, *Staphylococcus hominis*, and *Staphylococcus capitis*. A positive correlation was observed among *Staphylococcus saprophyticus*, *Lactobacillus lactis*, *Leuconostoc mesenteroides*, and *Clostridium saccharolyticum*. In contrast, *E. faecalis*, *B. licheniformis*, and *B. pumilus* were negatively correlated. *B. amyloliquefaciens* showed a negative correlation with *B. cereus*, *B. clausii*, *B. thuringiensis*, *Bacillus* sp. B14905, *Lysinibacillus sphaericusi*, and *Bacillus cytotoxicus.* Moreover, *L. lactis* was observed to be negatively correlated with *S. saprophyticus*, and *E. casseliflavus.*

### Genomic Signature of Bacteriophages

The metagenomic scaffolds were screened for the bacteriophage signatures using MetaPhinder. This analysis revealed a total of 54,002 scaffolds positive for bacteriophages sequences in the *Kinema* samples (Supplementary Table S6). A total of 174 bacteriophage taxa were identified in the four metagenomes. The taxonomical structure of *Kinema* metagenome phages was comprised of 1 order (*Caudovirales*), 4 families (*Siphoviridae*, *Myoviridae*, *Podoviridae*, and *Tectiviridae*), 34 genera, and 96 species (Supplementary Table S7). Further, many metagenomic sequences were mapped to the taxonomic terms assigned as “no-rank” in the taxonomic classification (Supplementary Table S7).

**FIGURE 4:**
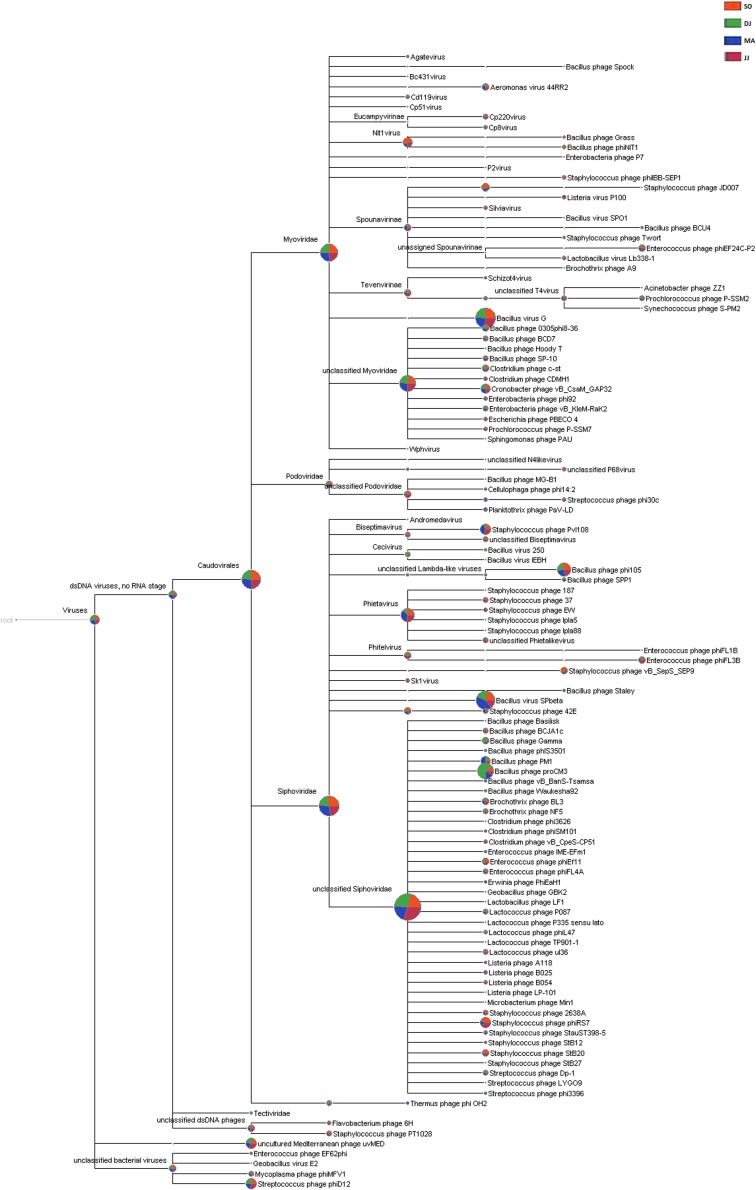
Comparative analysis of phage taxonomy in four *Kinema* samples, SO, DJ, MA, and JJ using MEGAN. Phages identified from *Kinema* samples are represented by different colors.

*Siphoviridae* was the most abundant family, followed by *Myoviridae*, and *Podoviridae* in all the four metagenomes. A few counts for the family *Tectiviridae* were also detected in the metagenome of DJ sample. At the genus level, the phages belonging to *Phietavirus*, *Nit1virus*, and *Kayvirus* were predominantly present in *Kinema* (Supplementary Table S7). Taxonomic distribution at species level revealed the dominance of bacteriophage specific to *Bacillus* species (*Bacillus virus G*, *Bacillus virus SPbeta*, *Bacillus phage proCM3*, and *Bacillus phage phi105*). DJ and MA samples showed a relatively higher representation of *Bacillus phage proCM3*, and *Bacillus phage SPbeta*, respectively. The well-known hosts for the above-identified phages could be *Bacillus megaterium*, *B. subtilis* and *B. pumilus, B. thuringiensis* and *B. subtilis*, respectively. Core Biome features analysis by Megan pipeline further confirmed the taxonomic profiling of bacteriophages in *Kinema* samples (Figure 4 and Supplementary Figure S7).

### Functional Feature Analysis

The mapping of metagenomic ORFs to the databases of orthologous gene groups (COG and KO) revealed many enriched functional features. Out of the total mapped ORFs, 38.65 to 47.88% were assigned to COG genes; whereas, 26.12 to 33.3% ORFs could be assigned as KEGG pathway genes. PCA analysis was performed to investigate the association among the KEGG functions of the seasonal *Kinema* samples. In PCA plot, the four metagenomic samples clustered separately. The functional variation was covered in two components- PC1 and PC2, with a maximum variance of 77.6% (Figure 3B).

COG mapping enriched the metagenomic ORFs into the functional features of cellular processes and signaling, information storage and processing, and metabolism (Figure 2A and Supplementary Table S2). Metabolism was the most predominant function, represented by 47.30 to 49.22% mapped ORFs in the *Kinema* samples collected in different seasons. At sub-level 2, a higher representation of general function prediction, ranged from 16.62 to 17.14%, followed by carbohydrate transport and metabolism (11.10 to 12.54%), and amino acid transport and metabolism (10.10 to 10.40%) in the seasonal *Kinema* metagenomes. The other COG categories were energy production and conversion (6.20 to 7.38%), inorganic ion transport and metabolism (6.35 to 6.59%), cell wall/membrane/envelope biogenesis (5.66 to 6.26%), translation, ribosomal structure and biogenesis (6.20 to 7.21%), replication, recombination, and repair (5.26 to 6.10%) (Figure 2B and Supplementary Table S2).

**FIGURE 5:**
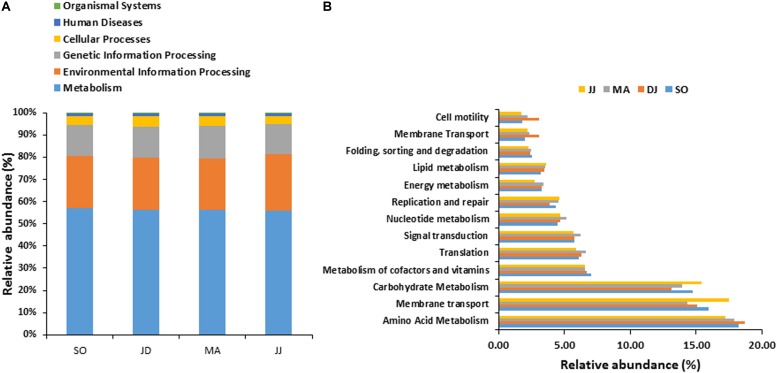
KO based mapping of metagenomics derived ORFs at level 1**(A)**, and level 2**(B)**. Top thirteen abundant phyla are shown for representation.

In KEGG annotation, metagenomic ORFs were aligned with the genes associated with a total of 145 metabolic pathways. Metabolism was the most abundant KEGG function, as found in COG analysis. KEGG mapping classified the metagenomic ORFs in metabolism (55.98 to 57.00%), environmental information processing (22.84 to 25.33%), genetic information processing (13.65 to 14.78%), cellular processes (3.53 to 5.01%), human diseases (1.36 to 1.49%), and organismal systems (0.16 to 0.20%) (Figure 5A and Supplementary Table S2).

At KO level-2, the top five abundant functions were- amino acid metabolism, membrane transport, carbohydrate metabolism, the metabolism of cofactors and vitamins, and translation (Figure 5B and Supplementary Table S2). Further analysis at subcategory level revealed the information about a majority of genes involved in glycine, serine and threonine metabolism (PATH: ko00260) (2.98 to 3.29%), alanine, aspartate and glutamate metabolism (PATH: ko00250) (2.25 to 2.86%), cysteine and methionine metabolism (2.15 to 2.41%) (ko00270), and arginine and proline metabolism (2.10 to 2.34%) (PATH: ko00330) (Supplementary Table S3). The genes related with valine, leucine and isoleucine biosynthesis (PATH: ko00280) (1.26 to 1.67%), histidine metabolism (PATH: ko00340) (1.35 to 1.56%), phenylalanine, tyrosine and tryptophan biosynthesis (PATH: ko00400) (1.17 to 1.34%), and lysine biosynthesis (PATH: ko00300) (1.09 to 1.33%) were also detected. A considerable representation of a key enzyme for glycine, serine and threonine metabolism, 2,3-bisphosphoglycerate-dependent phosphoglycerate mutase (K01834) (EC:5.4.2.1), was observed. The genes encoding enzymes catalyzing ectoine biosynthesis e.g., L-2,4-diaminobutyric acid transaminase (EctB) (EC:2.6.1.76), N_γ_-acetyltransferase (EctA) (EC:2.3.1.178), ectoine synthase (EctC) (EC:4.2.1.108), and ectoine hydroxylase (EctD) (EC:1.14.11), were also detected. These enzymes are typically encoded by *ectABCD* gene cluster of bacteria (García-Estepa et al., 2006; Bursy et al., 2008; Supplementary Figure S4 and Supplementary Table S4).

In the category of membrane transport, the genes related to ABC transporters (PATH: ko02010) (11.78 to 12.37%), and phosphotransferase system (PTS) (PATH: ko02060) (3.59 to 6.40%) were found in abundance (Supplementary Table S4). Furthermore, Dipeptide transport system, ATP-binding protein (K02035), cellobiose-specific IIC component (K02761), and beta-glucosides-specific IIB component (K0275) (EC: 2.7.1.69) were the abundant functions of ABC transporters and PTS system (Supplementary Figure S5).

Carbohydrate metabolism was the third most abundant category under metabolism, where a majority of the ORFs were mapped to the genes involved in pentose phosphate (PATH: ko00030) (1.94 to 2.34%), and glycolysis/gluconeogenesis (PATH: ko00010) (1.83 to 2.28%) pathways (Supplementary Table S3). A significant proportion of ORFs was mapped to the pathways of amino sugar and nucleotide sugar metabolism (PATH: ko00520) (1.50 to 1.93%), pyruvate metabolism (PATH: ko00620) (1.35 to 1.75%), and galactose metabolism (PATH: ko00052) (1.22 to 1.48%). The ORFs homologous to the genes of pentose and glucuronate interconversions (1.06 to 1.37%), fructose and mannose metabolism (1.13 to 1.19%), and citrate cycle/ TCA cycle (0.88 to 1.23%) metabolic pathways were noticed in the metagenome. The prevalent enzymes in pentose phosphate pathway were- 6-phosphogluconate dehydrogenase (K00033) (EC:1.1.1.44), ribokinase (K00852) (EC:2.7.1.15), 2-dehydro-3-deoxygluconokinase (K00874) (EC:2.7.1.45), and transketolase (K00615) (EC:2.2.1.1) (Supplementary Figure S6 and Supplementary Table S4).

The metabolism of the cofactors and vitamins were the third abundant subcategory of metabolism (Supplementary Table S4). The ORFs were mapped to porphyrin and chlorophyll metabolism pathway (ko00860) ranging from 1.09 to 1.36% in seasonal samples. In the category of translation, putative genes related to aminoacyl-tRNA biosynthesis (PATH: ko00970) (2.78 to 3.13%) were also identified.

### Carbohydrate Active Enzymes

To understand the dynamics of carbohydrate fermentation in *Kinema* by microbial enzymes, scaffolds were searched against the gene families of the CAZy database. A total of 2,781 sequences with catalytic domain for the carbohydrate-active enzyme (CAZyme) were identified in *Kinema* metagenomes at the criteria of 25% identity and 50% subject coverage cutoff (Table 2 and Supplementary Table S8). Putative CAZymes were distinguished from six CAZy families i.e., glycosyl hydrolases (GHs; 46.21 to 52.89%), glycosyltransferases (GTs; 30.85 to 32.70%), carbohydrate esterases (CEs; 9.58 to 13.16%), carbohydrate-binding modules (CBMs; 3.79 to 5.76%), auxiliary activities (AAs; 0.80 to 1.83%), and polysaccharide lyases (PLs; 1.73 to 2.00%).

**TABLE 2:** Summary of metagenomics scaffolds mapped to CAZy database.

**Cazy families**	**Putative CAZy genes ≥25% identity ≥50% subject coverage**			
	
	**SO**	**DJ**	**MA**	**JJ**
GH	340	481	269	265
GT	225	326	170	156
CE	71	137	64	48
CBM	29	60	26	19
AA	10	19	11	4
PL	13	18	11	9
Total	688	1041	551	501
		2781		

The taxonomic assignment of the putative CAZYme genes indicated that the majority of the sequences (86.77 to 91.29%) were contributed from Firmicutes bacteria. A relatively small percentage of CAZy sequences showed their belongingness to Proteobacteria (0.20 to 3.75%), Actinobacteria (2.54 to 7.41%), Thermotogae (0.29 to 1.0%), and Bacteroidetes (0.44 to 1.06%) (Supplementary Table S9). Interestingly, Firmicutes related sequences were found dominant in all the six CAZy families identified in the *Kinema* metagenomes of seasonal samples (SO, DJ, MA, and JJ). Proteobacteria majorly contributed for GTs in SO, DJ, and MA; whereas, GHs in JJ. On the contrary, Actinobacteria and Thermotogae were primarily associated with GHs in SO, DJ, MA, and JJ.

CAZy genes were further screened at more stringent criteria of ≥50% identity and ≥50% subject coverage in the metagenomic data (Table 3). The glycosidic bond hydrolyzing GHs were identified that could be involved in hemicellulose degradation (GH11, GH39, GH43, GH2, GH35, GH36, GH4, and GH42), cellulose branching/debranching (GH1, GH3, GH5, GH16, and GH31), starch branching/debranching (GH13), pectin branching/debranching (GH28, and GH78), polysaccharides degradation (GH26, GH38, GH51, GH53, GH92, GH185, and GH105), and oligosaccharide biosynthesis (GH32, GH68, and GH77) (Table 3). Among these, GH1 was found as the most abundant category, primarily represented by beta-glucosidase. The other predominant GH family enzymes were GH3 (beta-glucosidase), GH4 (α-galactosidase), GH13 (α-amylase), GH32 (levanase), and GH43 (beta-xylosidase). The hemicellulose degrading enzymes, 1,4-beta-xylanase, α xylosidase, and xylan 1,4-beta-xylosidase, were from GH11, GH31, and GH 39 families, respectively. The enzymes related to pectin modification were polygalacturonase (GH28), α-mannosidase (GH92), β-mannanase (GH26), rhamnogalacturonyl hydrolase (GH105). Some other prominent carbohydrate-related enzymes were chitinase (GH18), α-L-fucosidase (GH29), chitosanase (GH46), 4-α-glucanotransferase (GH77), glucuronyl hydrolase (GH88), and β-/α-galactosidase (GH2, GH35, and GH42).

**TABLE 3:** Carbohydrate active genes and their functions identified from CAZy database.

		**Putative CAZy genes in the four metagenomes (≥50% identity, ≥50% subject coverage)**			
		
**CAZY family**	**Annotations**	**SO**	**DJ**	**MA**	**JJ**
**Hemicellulose degradation**					
GH11	Endo 1,4-beta-xylanase	2	2	1	1
CE4	Polysaccharide deacetylase, Chitin deacetylase	15	53	17	10
GH39	Xylan 1,4-beta-xylosidase	1	1	0	1
GH43	Beta-xylosidase, arabinan endo-1,5-α-L-arabinosidase, α-N-arabinofuranosidase	9	21	8	13
CE14	Deacetylase	11	10	8	4
GH2	Beta-galactosidase	8	9	4	3
GH35	Beta-galactosidase	3	6	1	6
GH36	A galactosidase	4	8	5	4
GH4	A galactosidase	19	30	16	17
GH42	Beta-galactosidase, beta-galacturonidase	6	11	4	3
GH1	Beta-glucosidase	60	76	54	52
GH16	Beta-glucanase, endo-beta-1,3-1,4 glucanase	1	0	1	1
GH5	Endoglucanase, cellulase	2	2	1	1
GH3	Beta-glucosidase	12	21	11	9
AA3	Oxidoreductase	3	3	2	1
GH31	A xylosidase, α glucosidase	2	4	6	3
GH13	A amylase, glucan 1,6-α-glucosidase, trehalose synthase, neopullulanase	24	36	22	19
GT5	Glycogen/starch_synthase	1	3	1	2
CBM26	A -amylase	1	0	0	0
GH78	A -rhamnosidase	1	2	0	0
GH28	Polygalacturonase	3	3	3	1
PL1	Pectate lyase	3	5	3	1
PL3	Pectate lyase	1	2	1	1
PL8	Polysaccharide lyase family_8	1	2	0	0
PL11	Rhamnogalacturonan lyase	1	2	1	0
CE8	Pectinesterase	1	4	1	0
GH51	A -N-arabinofuranosidase	5	6	2	2
GH53	Arabinogalactan endo-1,4-beta-galactosidase	1	0	0	0
GT85	Arabinofuranosyl transferase	1	0	0	0
GH105	Rhamnogalacturonyl hydrolase	1	6	3	2
GT53	Arabinosyl transferase	1	0	0	0
GH92	A -1,2-mannosidase	2	4	0	3
GH38	A mannosidase	7	10	3	5
GH26	Beta-mannanase, mannan endo-1,4-beta-mannosidase	2	2	3	0
GT0	Mannosyltransferase	8	4	2	1
**Lipase/esterase**					
CE1	Tributyrin esterase	5	5	4	4
CE0	Hydrolase, GDSL-like Lipase	3	10	4	1
AA0	Multicopper oxidase	1	5	3	0
AA1	Multicopper oxidase	0	1	0	0
**Oligosaccharides production**					
GH32	Levanase, sucrose-6-phosphate hydrolase, sucrase-6-phosphate hydrolase	13	18	11	11
GH68	Levansucrase	2	0	1	1
**Others**					
GT4	D-inositol 3-phosphate glycosyltransferase, glycosyl transferase	42	49	36	29
GH18	Chitinase	3	4	2	0
GH29	A -L-fucosidase, α-1,3/4-fucosidase	4	3	2	6
GH46	Chitosanase	1	1	1	0
GH88	Glucuronyl hydrolase	5	6	3	7
GH77	4-α-glucanotransferase	1	0	0	0
CBM12	Chitinase	1	3	0	1
Total		304	453	251	226
			1234		

Glycosyltransferases (GTs) that are involved in the biosynthesis of disaccharides, oligosaccharides, and polysaccharides were the second most abundant CAZymes (Table 3). D-inositol 3-phosphate glycosyltransferase (GT4) was highly represented in the *Kinema* metagenomes (SO, DJ, MA, and JJ). Additionally, glycogen/starch synthase (GT5 and GT35), mannosyl transferase (GT 0), arabinosyl transferase (GT53), and arabinofuranosyl transferase (GT85) were also detected.

Carbohydrate binding modules (CBMs) were detected having an affinity for cellulose (CBM3, CBM20, and CBM48), starch (CBM25, CBM26, CBM34, CBM48, and CBM68), chitin (CBM5, and CBM12), and levan (CBM66). The families, CBM3, CBM20, and CBM48, were represented by cyclomaltodextrin glucanotransferase, endo-1,4-beta-glucanase (Cel5A), and pullulanase, respectively (Table 3). Further, α-amylase (CBM25, and CBM26), pullulanase (CBM48 and CBM68), and CBM34 enzymes, amylopullulanase, neopullulanase, and α-amylase were represented by the ORFs in the *Kinema* metagenome.

Carbohydrate esterases (CEs), catalyzing de-O or de-N-acylation of the substituted saccharides were identified that could be involved in hemicellulose degradation (CE4, and CE14), pectin degradation (CE8), and hydrolysis of lipids and esters (CE0, and CE1) (Table 3). The sequences mapped to CE4, and CE14 families were annotated as xylanase/chitin deacetylase, polysaccharide deacetylase, and deacetylase.

Auxiliary activities (AA), and Polysaccharide Lyases (PLs) family enzymes were relatively less represented in the metagenomes. Some of the metagenomic sequences were mapped on oxidoreductase, choline dehydrogenase (AA3), pectate lyases (PL1, and PL3), polysaccharide lyase (PL8), and rhamnogalacturonan lyase (PL11) (Table 3).

### Bioactive Potential

The bioactive potential of metagenomic samples was investigated by exploring genomic information about glutamic acid decarboxylase (GAD), and proteases (Supplementary Table S10). GAD catalyzes the decarboxylation of glutamate to γ-aminobutyric acid (GABA). A total of 12 genes were found associated with GAD (Supplementary Table S10). Some of the sequences exhibited 100% identity with GAD from *Enterococcus* sp. (WP_002309699, WP_003127278, WP_005233215, and WP_074934182.1), and *B. cereus* (WP_047385522, and WP_001135423). Putative homologous sequences were identified showing the identity of 50 to 84% with GAD protein sequences from *L. lactis*, *B. megaterium*, *Clostridium perfringens*, *Carnobacterium maltaromaticum*, and *E. coli* (Supplementary Table S10). Interestingly, the maximum representation of GAD associated genes was in DJ sample, in comparison to the samples from other seasons.

Proteases play an important role in the production of bioactive peptides during *Kinema* fermentation (Rai et al., 2017). Gene mining using the MEROPS database revealed the presence of proteases in *Kinema* metagenome. The *Kinema* metagenome identified a total of 73 proteases and 03 protease inhibitors in the metagenomic data (Supplementary Table S10). The protease associated metagenomic sequences were mapped to six families, aspartic proteases, cysteine proteases, glutamic proteases, metalloproteases, serine proteases, and threonine proteases. Among these, metallo, serine, and cysteine proteases were represented by a relatively higher number of enzymes (Supplementary Figure S8 and Supplementary Table S10). Further, the metagenome of SO exhibited relatively more representation of protease-related genes.

### Novel Biocatalyst Genes

Gene mining was done to distinguish genomic information about the novel enzymes for carbohydrate processing, and biosynthesis of bioactive compound production during *Kinema* fermentation. The metagenomic sequences exhibiting at least 20% difference (at protein level) and 90% subject coverage with the known genes in public protein database were explored for the identification of novel genes. Novel putative genes encoding β-galactosidase, β-glucosidase, β-xylosidase, and GAD were identified in the metagenome (Table 4). The metagenomic beta-galactosidase shared 57−70% identity at the protein level with GH2 and GH35 family genes from *Enterococcus aquimarinus* (WP_071874669), *Enterococcus phoeniculicola* (WP_010769467), and *Enterococcus* sp. (WP_123866866) (Supplementary Figure S9). The putative beta-glucosidase exhibited 62% identity with a gene from *Carnobacterium maltaromaticum* (WP_101703675). The metagenomic beta-xylosidase was 64% identical to WP_075420282.1 (*megaterium*). GAD associated metagenomic sequences showed 21−26% difference with the known genes from *Carnobacterium maltaromaticum* (WP_035065015, WP_010052159, WP_035065015, and WP_010052159.1). The domain architectures of these genes have been represented in Supplementary Figure S9.

**TABLE 4:** Annotation of novel genes involved in carbohydrate processing and γ-aminobutyric acid (GABA) production.

	**NR database**				**CDD**		
		
	**Query id**	**Description**	**Identity**	***E*-value**	**Annotation**	**Interval**	***E*-value**
Beta-galactosidase	DJ_Scaffold_4422	WP_123866866.1 glycoside hydrolase family 2 (*Enterococcus* sp. FDAARGOS_553)	69.85%	0	cl27651 Glycosyl hydrolases family 2	17−440	1.68E-40
	JJ_Scaffold_4305	WP_071874669.1 beta-galactosidase (*Enterococcus aquimarinus*)	56.54%	0	pfam01301 Glycosyl hydrolases family 35	8−326	2.53E-141
	MA_Scaffold_3895	WP_010769467.1 hypothetical protein (*Enterococcus phoeniculicola*)	64.31%	0	cl27651 Glycosyl hydrolases family 2	22−440	4.31E-39
Beta-glucosidase	MA_Scaffold_7958	WP_101703675.1 glycosyl hydrolase (*Carnobacterium maltaromaticum*)	61.61%	0	cl27622 Glycosyl hydrolase family 3 N terminal domain	33−647	6.50E-90
Beta-xylosidase	JJ_Scaffold_2825	WP_075420282.1 glycoside hydrolase family 43 protein (*Bacillus megaterium*)	64.01%	0	cd09000 Glycosyl hydrolase family 43, beta-D-xylosidase	6−317	1.28E-141
Glutamate decarboxylase	SO_Scaffold_3270	WP_010052159.1 glutamate decarboxylase (*Carnobacterium maltaromaticum*)	74.11%	0	cl18945 Aspartate aminotransferase (AAT) superfamily (fold type I) of pyridoxal phosphate (PLP)	12−429	0.00E+00
	MA_Scaffold_115	WP_035065015.1 glutamate decarboxylase (*Carnobacterium maltaromaticum*)	78.70%	0	cl18945 Aspartate aminotransferase (AAT) superfamily (fold type I) of pyridoxal phosphate (PLP)	1−378	0.00E+00
	JJ_Scaffold_12355	WP_010052159.1 glutamate decarboxylase (*Carnobacterium maltaromaticum*)	77.11%	0	cl18945 Aspartate aminotransferase (AAT) superfamily (fold type I) of pyridoxal phosphate (PLP)	14−438	0.00E+00
	DJ_Scaffold_7824	WP_035065015.1 glutamate decarboxylase (*Carnobacterium maltaromaticum*)	74.11%	0	cl18945 Aspartate aminotransferase (AAT) superfamily (fold type I) of pyridoxal phosphate (PLP)	11−429	0.00E+00

The DNA fragments encoding, β-galactosidase, β-glucosidase, β-xylosidase, and GAD, were PCR amplified from the *Kinema* metagenome, using the gene-specific primers (Supplementary Figure S10 and Supplementary Table S11), followed by gene sequencing. The gene sequences of metagenomic β-galactosidase, β-glucosidase, β-xylosidase, and GAD were used for three-dimensional structural prediction, following the homology modeling approach, using the pdb templates, c3lpgA, c5wabD, c1yifC, and c5gp4C, respectively. On superimposition of the homology models over their template, the root-mean-squared-deviation (RMSD) values were obtained in the range of 0.032 to 0.203 Å (Supplementary Figure S11).

**FIGURE 6:**
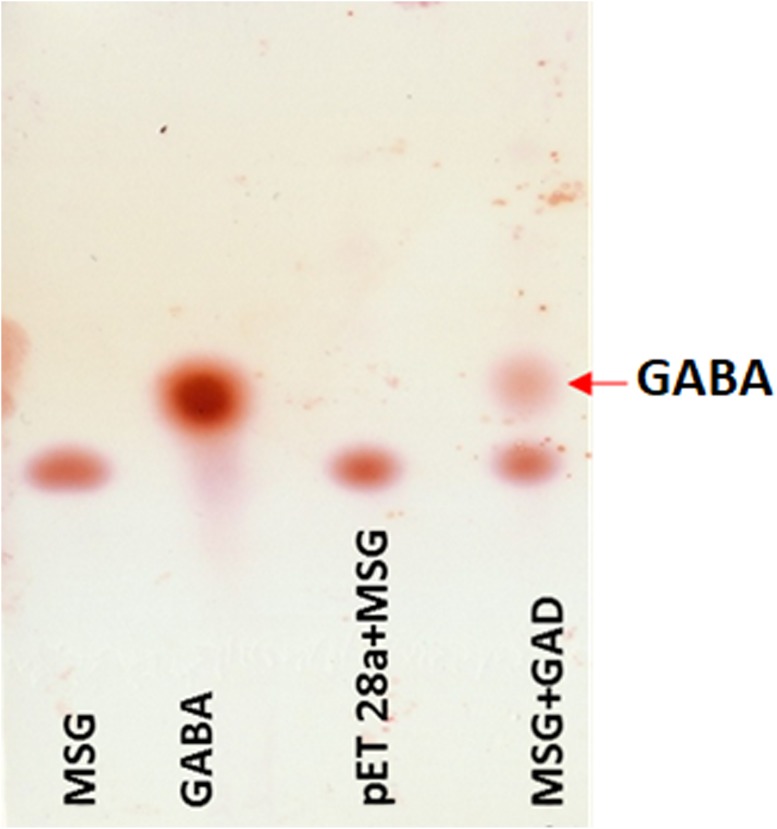
Thin layer chromatography analysis of GAD activity. Reactions containing standard of 1 uL MSG (10 mM), 0.5 uL GABA (10 mM), 1 uL control (pET28a and MSG), and 1 uL of GAD incubated with 10 mM MSG spotted in lane 1 to 4, respectively, are shown.

The putative *GAD* gene was expressed in *E. coli* BL21 (DE3). The crude cell-extract of un-induced *E. coli* and GAD expressing cells were loaded in SDS-PAGE gel. PAGE analysis confirmed substantial expression of GAD in soluble fraction (Supplementary Figure S12). The MSG substrate was treated with the crude cell-extract of GAD expressing *E. coli*. As a control, a reaction was performed with MSG substrate, taking crude cell-extract of vector transformed cells. TLC analysis of GAD treated MSG showed the spots for GABA in the reaction. The control reaction did not show any spot for GABA (Figure 6). This result validated the catalytic function of the novel GAD gene identified in the *Kinema* metagenomic resource.

## Discussion

The soybean fermented food product, *Kinema*, is being consumed as a nutritional supplement, since last several years by the local tribal population inhabiting Sikkim Himalaya region. Fermentation is a complex microbial process that plays a pivotal role in the biotransformation of complex biomolecules to the simpler and bioactive forms. The traditional fermented product, *Kinema*, represents a synergistic action of microbial enzymes for the transformation of macro-bio-molecules, i.e., carbohydrates, proteins, and polyphenols (Rai et al., 2017). The objective of the present study was to analyze the taxonomic and functional profiles of fermented soybean *Kinema* samples collected during different seasons, and exploration of the genomic resource of desirable biocatalysts for value-added biomolecules.

The excessive predominance of firmicutes, and the presence of actinobacteria and proteobacteria as minority groups in *Kinema* metagenome, are consistence with the previous findings on the fermented soybean food products, e.g., *douchi* (Yang et al., 2016), and *doenjang* (Jung W.Y. et al., 2016). *Bacillus* from Bacillales, and *Enterococcus* from Lactobacillales were inferred as the major player to execute the fermentation process in Kinema. The higher representation of three *Bacillus* sp., *B. subtilis*, *B. amyloliquefaciens*, and *B. licheniformis*, in the metagenomic profile coincided with the microbial population analysis of soybean fermented food products in the previous reports (Kubo et al., 2011; Oguntoyinbo et al., 2007; Rai et al., 2017). *Bacillus* sp. secretes extracellular enzymes involved in the enzymatic transformation of carbohydrates and protein macromolecules, the during fermentation (Rai et al., 2017). The mucilage produced by *Bacillus* species gives the preferred stickiness property to *Kinema* (Chettri et al., 2016).

The presence of amino-acid producing bacteria, *B. thuringiensis*, and *Corynebacterium glutamicum* (Hibi et al., 2011; Hahne et al., 2018), is in line with the nutritive significance of *Kinema*. The metagenomic profile of *Kinema* revealed genomic clues about the microorganisms that have been reported to exhibit probiotic properties, such as, *L. lactis*, *B. subtilis*, and *B. clausii* (Canani et al., 2007; Kechagia et al., 2013). Another abundant genus, *Enterococcus*, is being considered to have probiotic potential and biosynthetic capability of natural antimicrobial agents in food products (Hanchi et al., 2018). The species, *E. faecium*, *E. casseliflavus*, and *E. faecalis*, have been suggested to be useful microbes in fermentation (Franz et al., 2007; Corsetti et al., 2012). However, this genus has not been yet accorded generally recognized as safe (GRAS) status, and its safety concerns need to be completely resolved.

Uncontrolled fermentation sometimes leads to growth of undesirable bacterial population. Metagenome analysis detected the presence of *B. cereus*, which is a food-borne pathogenic bacterium reported earlier in fermented soybean product (Jeyaram et al., 2008). Apart from *B. cereus*, the possible pathogenic microorganisms in *Kinema* could be *S. saprophyticus*, *P. mirabilis*, *P. penneri*, *L. monocytogenes*, and *S. aureus* (Farber and Peterkin, 1991; Jeyaram et al., 2008; Kishore, 2012; Ehlers and Merrill, 2019). Interestingly, the dominance of these undesirable bacterial species also varied with the seasonal difference, which could be due to temperature variation (Roy et al., 2011; Jung J.Y. et al., 2016). Increase in the abundance of *B. cereus*, *P. mirabilis*, and *P. penneri* during DJ was marked by a relative decrease in the abundance of *S. saprophyticus*, and *S. aureus*. Among the unpleasant bacteria present in *Kinema*, *Staphylococcus saprophyticus*, *P. mirabilis*, and *P. penneri* have been reported as causative agents of urinary tract infection (UTI) (Kishore, 2012; Ehlers and Merrill, 2019). Unfortunately, *Proteus* sp., *E. faecium*, and *S. aureus*, detected in *Kinema* metagenome, are included in the list of critical and high-priority antibiotic-resistant bacteria (WHO, 2017). The genomic marks of these nasty bacterial species may indicate about foodborne illness related agents in *Kinema*. The degree of harmfulness of these strains from *Kinema* needs to be studied. However, the secretion of antimicrobial compounds by *B. subtilis*, *B. pumilus*, *B. licheniformis*, and Lactic acid bacteria could be the efficient biomolecules in *Kinema* to work against the growth of the unwanted microorganisms during fermentation (Ouoba et al., 2007; Abriouel et al., 2011). This was supported by the observation of negative correlation among beneficial candidates (such as *B. licheniformis*, *B. pumilus*, *B. amyloliquefaciens*, and *L. lactis*), and the unpleasant bacteria including the opportunistic species (for example *B. cereus*, *B. cytotoxicus*, *S. saprophyticus*, *E. faecalis*, *B. clausii*, *B. thuringiensis*, *L. sphaericus*, and *E. casseliflavus*) identified in *Kinema* metagenome. This could be suggestive of application of these beneficial microbes in food preservation. Further, a controlled fermentation with the selective microbes in *Kinema* can be a desirable way to avoid the growth of bacterial pathogens.

The dominance of viral families like *Siphoviridae*, *Myoviridae*, and *Podoviridae*, belonging to the order *Caudovirales*, in the *Kinema* metagenome is in consistence with the previous report on fermented shrimp, sauerkraut, and kimchi (Park et al., 2011). Our findings indicated that the community of bacteriophages present in *Kinema* were less diverse as compared to the different reports on the environmental samples (Luo et al., 2017; Graham et al., 2019). Bacteriophages are known to play a fairly important role in determining the relative abundance of bacterial communities over a period of time (Shapiro et al., 2010). The presence of bacteriophages related to the bacterial hosts belonging to *Bacillus*, *Staphylococcus*, *Enterococcus*, *Lactococcus*, and *Streptococcus* suggests their role in regulating the taxonomic diversity during *Kinema* fermentation (Gao et al., 2018). The study presents the dominance of bacteriophages and their targets against the beneficial and sickening bacterial species in *Kinema*, which possibly act as biocontrol agents. However, a detailed investigation is required to establish a host-phage relationship during *Kinema* fermentation.

The differential association among metagenomic samples was corroborated by the PCA plot analysis, performed on the basis of their taxonomic and functional features. The statistical evidence indicated the distinction in microbial load as well as the functional potential of the *Kinema* samples from the four seasons.

The functional profiling of the metagenomes suggested upregulation of amino acid metabolism, membrane transport, and carbohydrate metabolism pathways throughout the fermentation periods; this result provides the molecular evidence of protein and carbohydrate fermentation by the microbial community in *Kinema*. The comparable relative abundance of the function “metabolism,” as inferred by COG and KO mapping, reflects an active metabolism prevailed throughout the fermentation.

The metagenomic studies of *Kinema* fermentation identified glycine, serine, and threonine metabolism as the abundant pathway enzymes for ectoine biosynthesis. A variety of bacterial and a few archaeal microbes are known to produce a selective class of compatible solute, ectoine, and its hydroxylated derivative, i.e., hydroxyectoine, in order to cope with the high osmotic conditions, and stresses in their surroundings (Kuhlmann et al., 2011; Czech et al., 2018). The metagenomic sequences of only JD were mapped to ectoine biosynthetic pathway genes, which could be due to imbalances in osmolality encountered during the transition of temperature and moisture for a longer period of fermentation (Czech et al., 2018).

The abundance of genes involved in the metabolism of alanine, aspartate, glutamate, arginine, and proline supports the production of flavoring and aromatic compounds during fermentation (Sulaiman et al., 2014). Glutamate is used as a precursor molecule for the synthesis of γ-aminobutyric acid (GABA). Its role in the regulation of the nervous system is well defined (Hyland and Cryan, 2010; Xu et al., 2017). The presence of genes for the biosynthesis of branched-chain amino acids (BCAAs: valine, leucine, and isoleucine) were identified in our metagenome, which might be involved in influencing the genes involved in the metabolism of nitrogen or carbon in *B. subtilis* (Shivers and Sonenshein, 2004; Belitsky, 2015). A significant abundance of the genes involved in the metabolism of essential amino acids (e.g., methionine, histidine, lysine, valine, leucine, and isoleucine) reflects the rich nutritional value of *Kinema* (Watanabe et al., 2007; Dajanta et al., 2011). The results are in accordance with the previous studies demonstrating the production of essential free amino acids during soybean fermentation using *B. subtilis* strain isolated from *Kinema* (Sanjukta et al., 2015). The genes involved in the metabolism related to cofactors and vitamins are required for the functioning of enzymes, cellular metabolism, which finally improve the nutritional quality of the fermentation-derived product. In the *Kinema* metagenome, the genes mapped to these metabolisms could be contributed by *Lactococcus*, *Enterococcus*, and *Streptococcus* genera (O’Connor et al., 2005; Cooke et al., 2006; Turpin et al., 2011).

Peptide transport is involved in diverse cellular responses, including nutrition, signaling, gene expression regulation, sporulation, competence, and chemotaxis (Doeven et al., 2005). The dipeptide transport system ATP-binding protein (dppF) is an ABC transporter and a part of the DPP system, which facilitates the selective uptake of di- and tripeptides (Doeven et al., 2005). The high abundance of genes related to peptide transport, dipeptide transport system ATP-binding protein (dppF) reflects the requirement of microbial consortia surrounded by protein-rich niches in *Kinema*. Phosphoenolpyruvate (PEP): carbohydrate phosphotransferase system (PTS) is the source of transport and phosphorylation of various sugar forms such as monosaccharides, disaccharides, amino sugars, polyols, and other sugar derivatives in bacteria (Deutscher et al., 2006). The metagenome identified a large number of components for cellobiose-specific PTS, which help in the uptake of cellulose-derived products, including Glucose, β-glucosides, mannose, lactose, and fructose. The identification of CAZymes, involved in transforming the plant cell wall components, and the PTS systems, support the active role of the microbial population in the transformation of polysaccharide and short-chain carbohydrate during *Kinema* fermentation.

The abundance of carbohydrate metabolism (Pentose phosphate pathway, and glycolysis) identified in our *Kinema* metagenome is important for microbial metabolism (Sulaiman et al., 2014). The abundance of genes identified in galactose metabolism (α-glucosidase, α-galactosidase, and β-galactosidase) might be involved in the degradation of starch, and oligosaccharides content of soybean into simpler forms of sugar during fermentation. *Kinema* metagenome displayed the diverse set of CAZYmes that could be interactive with the carbohydrate, which constitutes 35% of soybean seeds (Karr-Lilienthal et al., 2005; Hou et al., 2009). The GH and GT family enzymes, which could be belonging to the phylum Firmicutes and Proteobacteria, respectively, perform an interplay in the processing of carbohydrate macromolecules during fermentation (Park et al., 2018). This results in the biosynthesis of disaccharide, oligosaccharide, and glycoconjugates (Van Laere et al., 2000; Breton et al., 2006). The *Kinema* metagenome was found to be equipped with the genes involved in the processing of lignocellulose (cellulose, hemicellulose, and pectin), which suggest that the plant-derived carbohydrate is the source of energy for aerobic (via tricarboxylic acid, TCA cycle) and anaerobic (fermentation) microbes (Mhuantong et al., 2015). GH5 and GH16 family cellulases, detected in the metagenome, could be involved in producing random internal cut within cellulose fiber, releasing the cellooligosaccharides (Martinez et al., 2008). The metagenomic GH1 and GH3 family β-glucosidases could be involved in the hydrolysis of β-1,4-linkages in the cellooligosaccharides (Wilkens et al., 2017), or participate in the pathway of soybean isoflavone glycosides biosynthesis (Cao et al., 2018). As other enzymes for cellooligosaccharides processing could not be detected in the metagenome, there is a possibility of the involvement of the abundant β-glucosidases in the biosynthesis of isoflavones in *Kinema*.

The presence of GH4 and GH36 family α-galactosidases in the metagenome is suggestive of the hydrolysis of α-galactosidic residue containing macromolecules present in soybean seeds (e.g., raffinose-series oligosaccharides, stachyose, raffinose, and verbascose) during the *Kinema* fermentation (Švejstil and Musilová Rada, 2015). Thus, α-galactosidases improves digestion, and in that way is helpful in reducing the flatulence issue associated with the consumption of soybean (Švejstil and Musilová Rada, 2015).

Xylans are the second most abundant polymer found in hemicellulosic material. The structure of xylan is a complex polymer that requires the combined activity of endoxylanase, xylosidase, and other enzymes for its hydrolysis or chemical modifications (Kane and French, 2018). In *Kinema* metagenome, xylan hydrolyzing enzymes were assigned to GH11 (Endo 1,4-beta-xylanase), GH39 (Xylan 1,4-β-xylosidase), and GH 43 (β-xylosidase), similar to the metagenomic profile of previously reported studies on the lignocellulosic material (Mhuantong et al., 2015; Wang et al., 2016). Further, the presence of deacetylase enzyme (CE4, and CE14) in *Kinema* metagenome might help in increasing the susceptibility of xylan to these hydrolytic enzymes (Pawar et al., 2017).

Amylose is a linear polymer of glucose residue joined by α-1,4-linkages, whereas, amylopectin is a branched polymer of amylose chains connected by α-1,6 linkages. Pullulan is a linear polymer of maltotriose units in which three glucose units are connected by an α-1,4 glycosidic bond, and two maltotriose units are connected to each other by an α-1,6 glycosidic bond. The metagenome represented the abundance of GH13 amylases, which act on α-1,4-linkages, suggesting starch as a possible source of glucose for microbial metabolism. Further, the association of GH13 family α-amylase and pullulanase with starch binding domains (CBM25, CBM26, and CBM34) might increase the substrate affinity during *Kinema* fermentation (Peng et al., 2014).

Pectin is a family of complex hetero-polysaccharide rich in galacturonic acid residues present in the plant’s cell wall (Voragen et al., 2009; Wang et al., 2016). The network of pectin contains 17 different monosaccharides and more than 20 different linkages (Bonnin et al., 2014). Degradation of pectin network requires specific and synergistic action of enzymes such as pectate lyase (PL1, PL3), polygalacturonase (GH28), arabinofuranosidase (GH51), rhamnogalacturonyl hydrolase (GH105), which were weakly represented in *Kinema* metagenome.

The presence of GH68 family levansucrase and GH32 family levanase indicate biosynthesis of levan derived prebiotic fructooligosaccharides during *Kinema* fermentation. This result is in agreement with the previous reports displaying levan, apart from Poly-γ-Glutamic Acid (PGA), as a major fraction of fermented soybean mucilage (Xu et al., 2006; Chettri et al., 2016). Raffinose and stachyose molecules, which are known to be rich in soybean product, can also be catalytically transformed into prebiotic carbohydrate by levansucrase (Jadaun et al., 2019).

Fermented soybean products are rich in a wide range of bioactive compounds responsible for health benefits (Cho Y.S. et al., 2011; Sanjukta and Rai, 2016). These compounds are either produced by starter culture or by the transformation of soybean components (proteins, polyphenols, and carbohydrates) into their bioactive form. Proteases are important enzymes responsible for the production of bioactive peptides (Rai et al., 2017). Soybean-derived bioactive peptides, produced during fermentation, exhibit several health benefits depending on their size, sequence, and composition of amino acids (Sanjukta and Rai, 2016). The type of bioactive peptides produced during soybean fermentation depends on the starter culture at the strain level, which specifically hydrolyzes soybean proteins (Sanjukta and Rai, 2016). The diversity among proteases in *Kinema* samples of different seasons suggests the possibility of finding novel bacterial strains suitable for bioactive peptide synthesis. Fibrinolytic enzymes are a group of serine proteases that are produced by the microorganisms associated with Asian fermented food products. They are therapeutically important enzymes useful in the treatment of thrombosis (Mine et al., 2005). Representation of serine proteases was higher in SO suggesting the possibility of a higher diversity of fibrinolytic enzymes during this period. Selection of microbial starter rich in specific proteases with high-efficiency of fibrinolytic enzymes can be applied for the production of fermented soybean product rich in bioactive peptides. The enzymes related to carbohydrate metabolisms, such as α-amylase and β-glucosidase, are known for bioconversion of bound polyphenols to their free form, which results in enhancing their bioactivity associated with soybean polyphenols (Rai et al., 2017). These enzymes were found abundant throughout the year, reflecting that soybean consumed in the different seasons may have similar polyphenol content. However, variation in the representation of GAD and other proteases suggest that naturally fermented soybean product of different seasons might have different types of bioactive compounds, resulting in variability of health benefits. The presence of a range of bioactive compounds may depend on the microbial strains dominant during that period of fermentation. Fermented product of specific or multiple health benefits can be developed by screening the beneficial isolates from different seasons.

## Conclusion

The metagenomic study represents the taxonomic and functional features of *Kinema* collected during different seasons. This study generated the genomic resource for potential biocatalysts for carbohydrate transformation and bioactive molecule production. Identification of diverse kinds of genes for carbohydrate-active enzyme and bioactive compound production expands the understanding of microbial bioprocesses during fermentation. This enhances our knowledge about the enzymatic processing events during the natural processing of soybean fermentation. The metagenomic study represents the taxonomic profiling in the *Kinema* samples from four different seasons, revealing the information about the prevalence of several beneficial and u microorganisms. The data of taxonomic and functional feature analysis of the seasonal *Kinema* samples can be further utilized for the designing of a starter culture, targeted to specific or multifarious health benefits, through controlled fermentation under hygienic conditions.

## Data Availability

The metagenomic raw reads used for this study were deposited in publicly accessible NCBI’s Sequence Read Archive (SRA) under the accession number: PRJNA529966.

## Author Contributions

SPS and AR designed and supervised the study. JK, NS, and GK performed all the experiments. SS and DS helped in the sample collection and data analysis. JK, SPS, and AR performed all the data analysis and prepared the manuscript.

## Conflict of Interest Statement

The authors declare that the research was conducted in the absence of any commercial or financial relationships that could be construed as a potential conflict of interest.

## Footnotes

1 http://www.illumina.com/

2 https://metagenomics.anl.gov/

3 https://biit.cs.ut.ee/clustvis/

4 https://www.xlstat.com/

5 https://cge.cbs.dtu.dk/services/MetaPhinder/

6 https://ab.inf.uni-tuebingen.de/software/megan6

7 http://www.cazy.org/

8 https://www.ncbi.nlm.nih.gov/

9 http://computationalbiology.ufpa.br/gofeat/

10 https://www.ncbi.nlm.nih.gov/refseq/

11 https://www.ebi.ac.uk/merops/
