# Supplementary material for: Metagenomic Insights Into the Taxonomic and Functional Features of Kinema, a Traditional Fermented Soybean Product of Sikkim Himalaya
Source: Front Microbiol. 2019 Aug 2;10:1744. doi: 10.3389/fmicb.2019.01744 (PMC6688588; doi:10.3389/fmicb.2019.01744)
Supplement: Supplementary file 12 [file Data_Sheet_1.PDF]

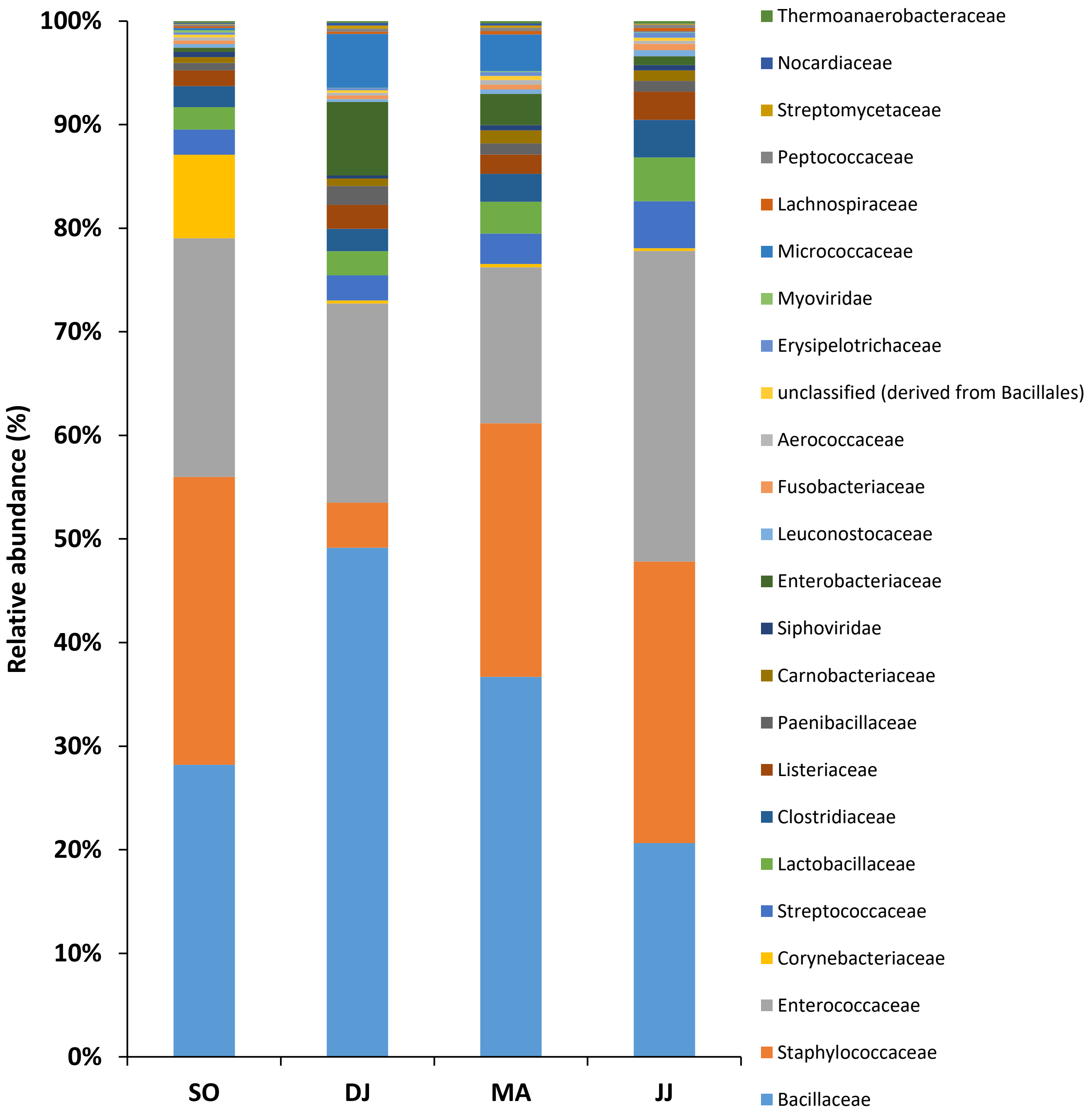

Supplementary Figure S1. Taxonomic abundance of microbial community at the rank of family level.

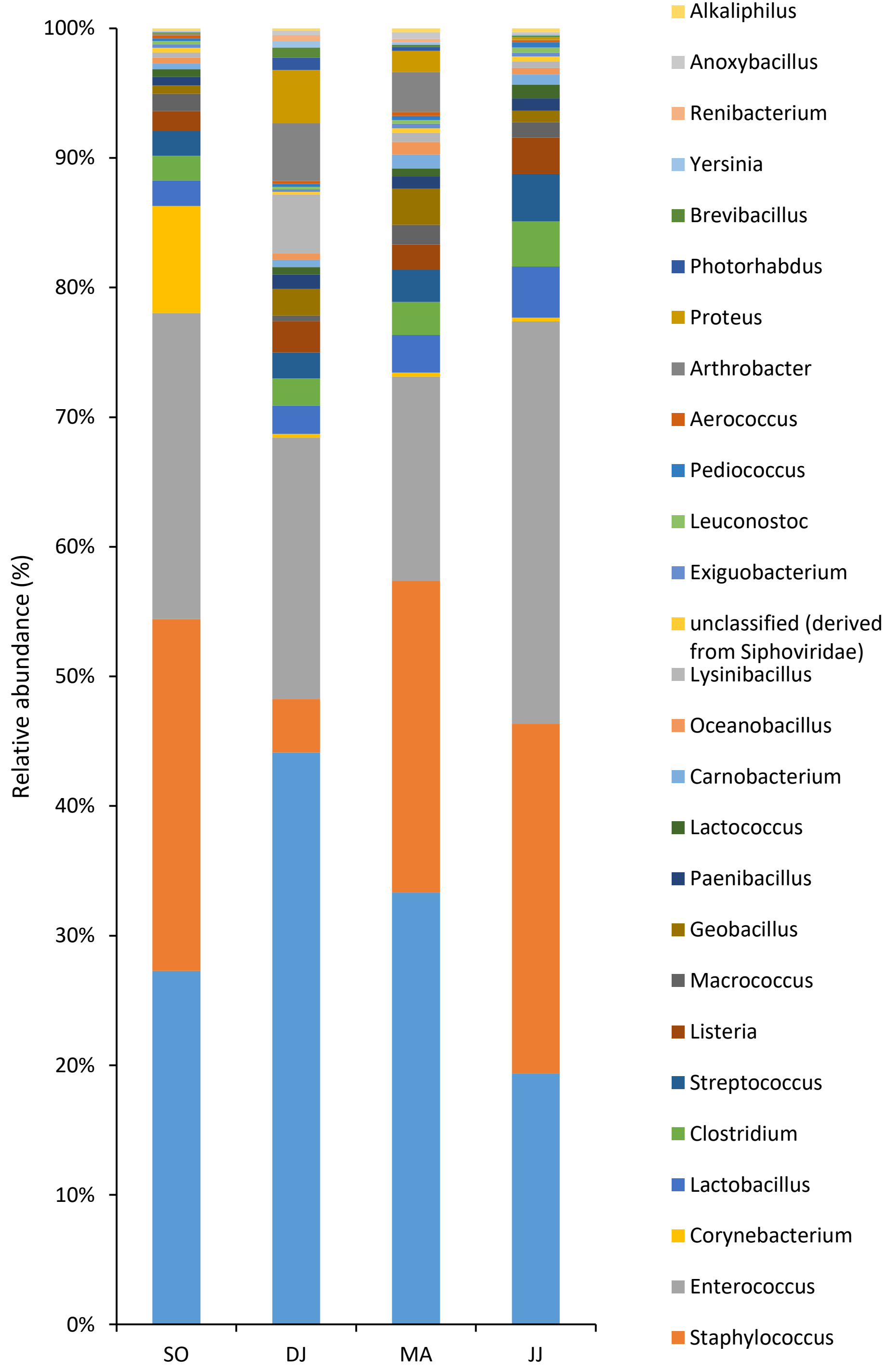

Supplementary Figure S2. Taxonomic abundance of microbial community at the rank of genus level.

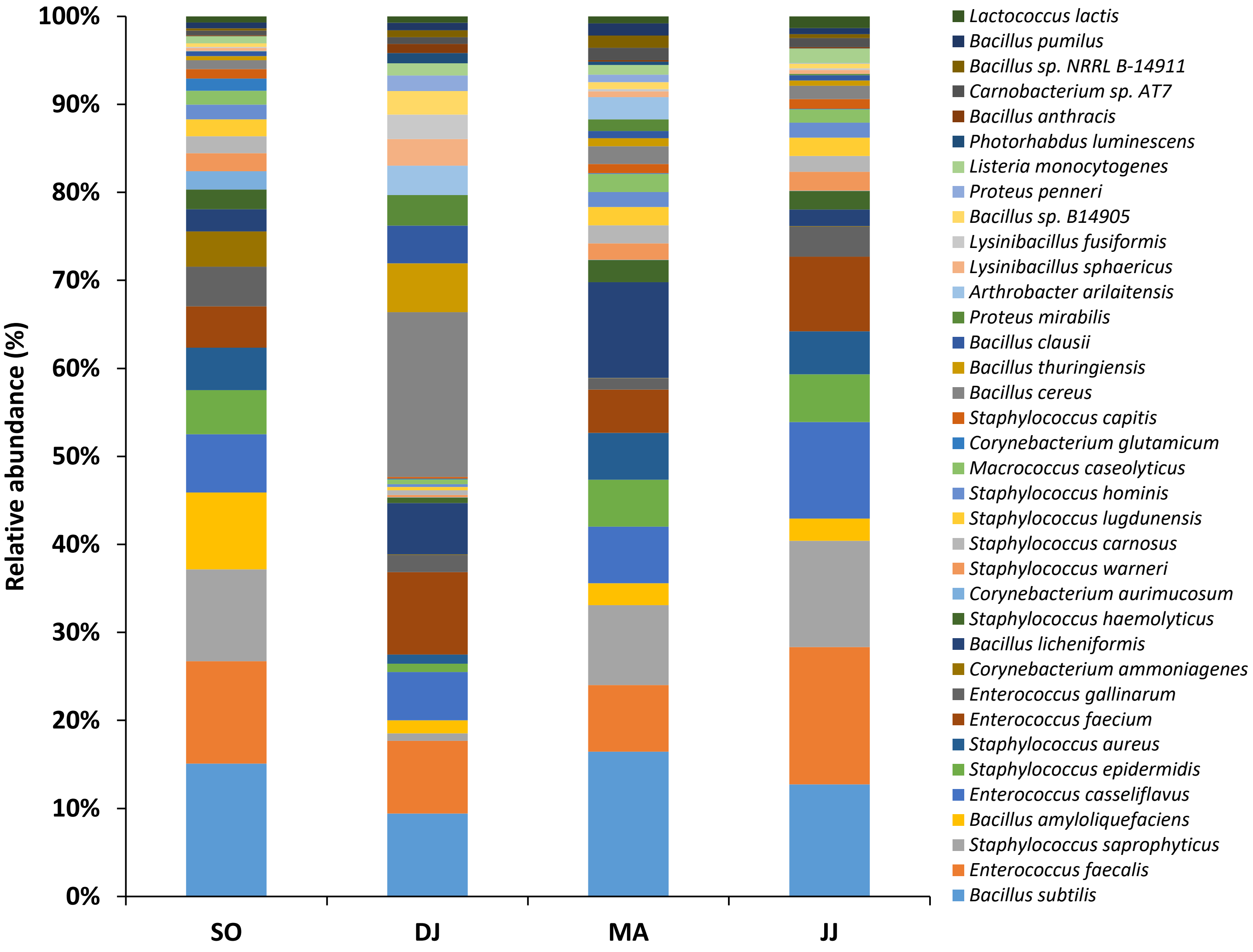

Supplementary Figure S3. Taxonomic abundance of microbial community at the rank of species level.

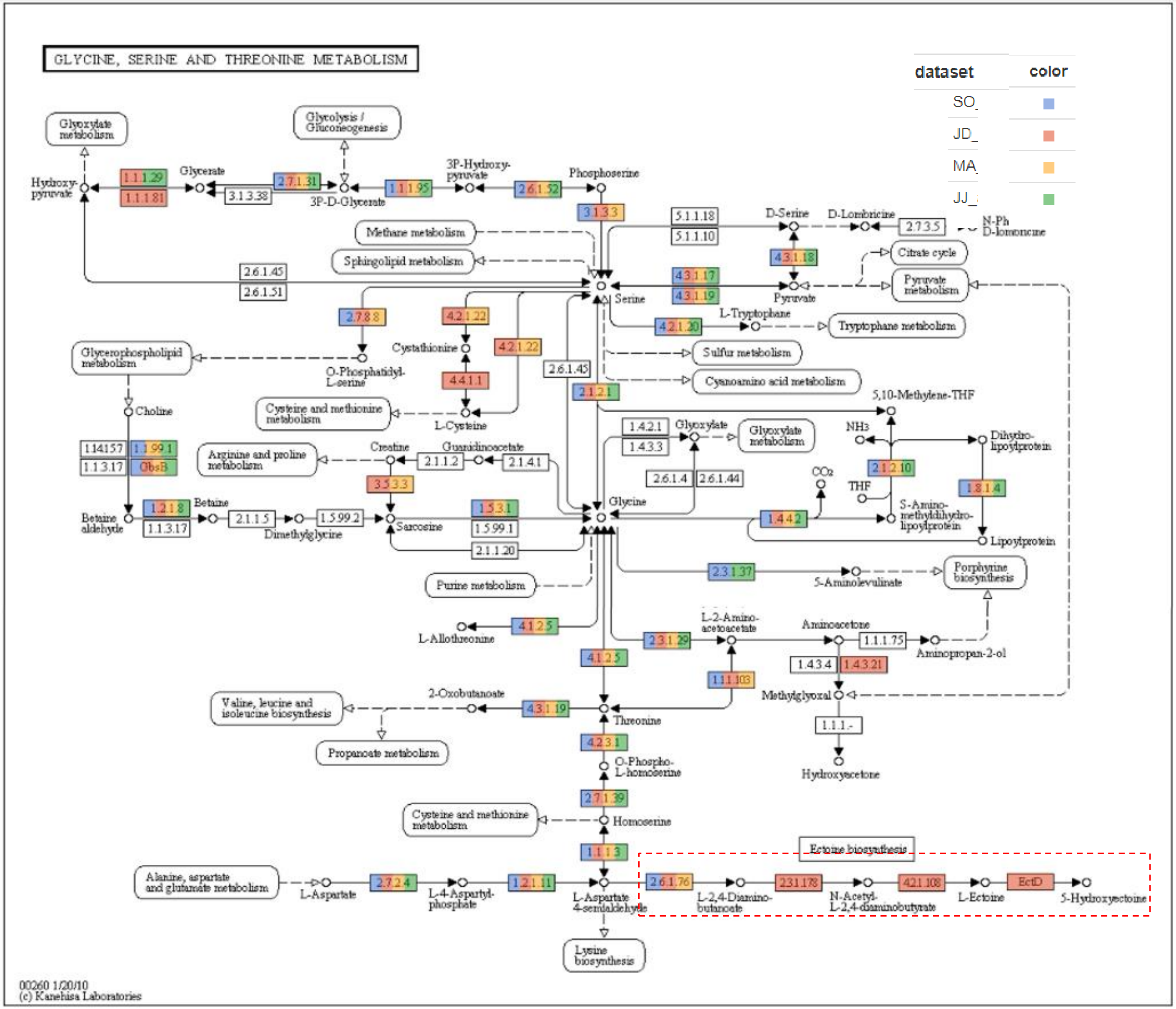

Supplementary Figure S4. KEGG pathway of glycine, serine and threonine metabolism identified from *Kinema* samples. The enzymes of kinema samples (SO, DJ, MA, and JJ) mapped in pathway are represented by different colors.

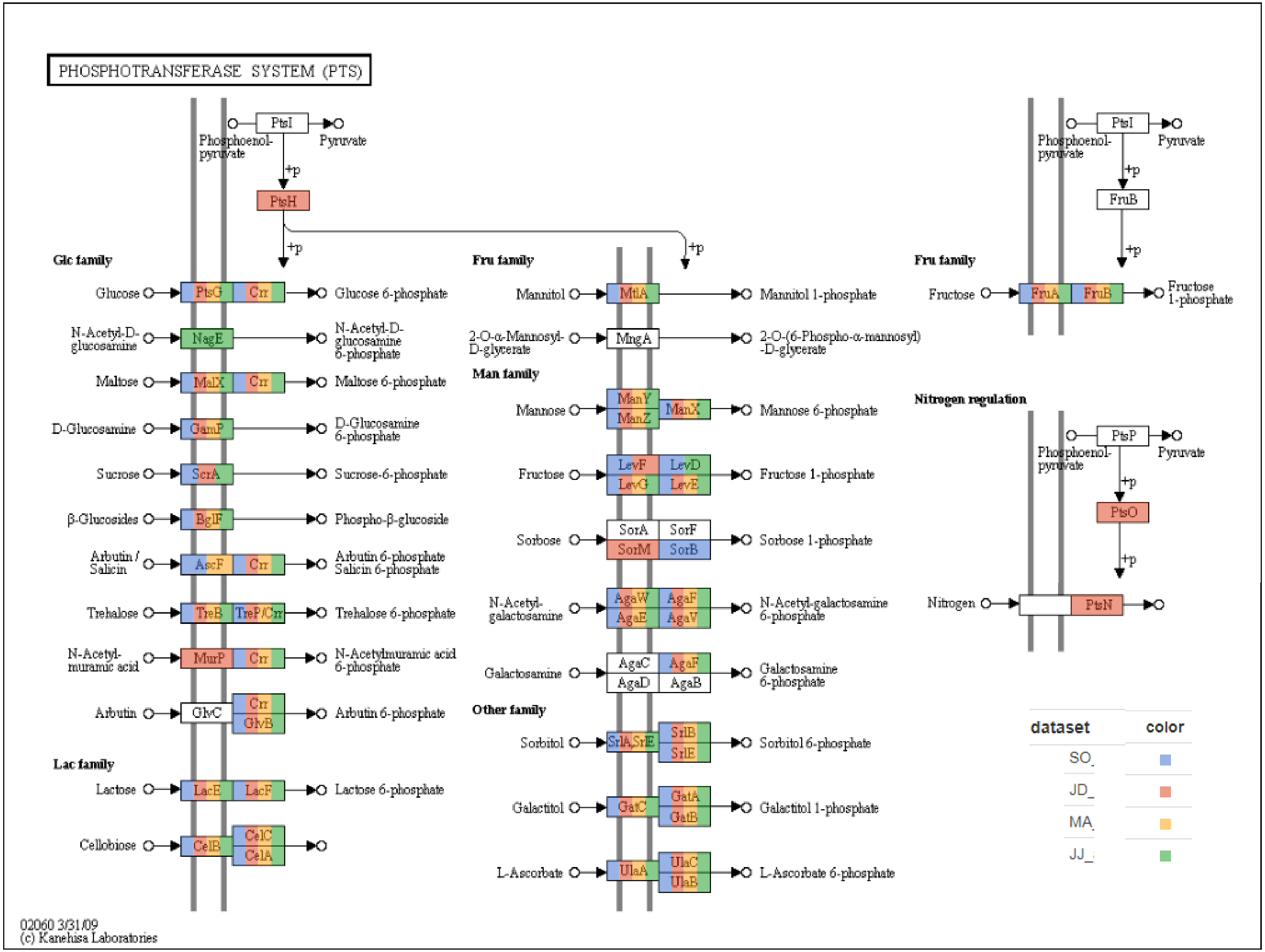

Supplementary Figure S5. KEGG pathway of phosphotransferase system (PTS) in kinema samples. The enzymes of kinema samples (SO, DJ, MA, and JJ) mapped in pathway are represented by different colors.

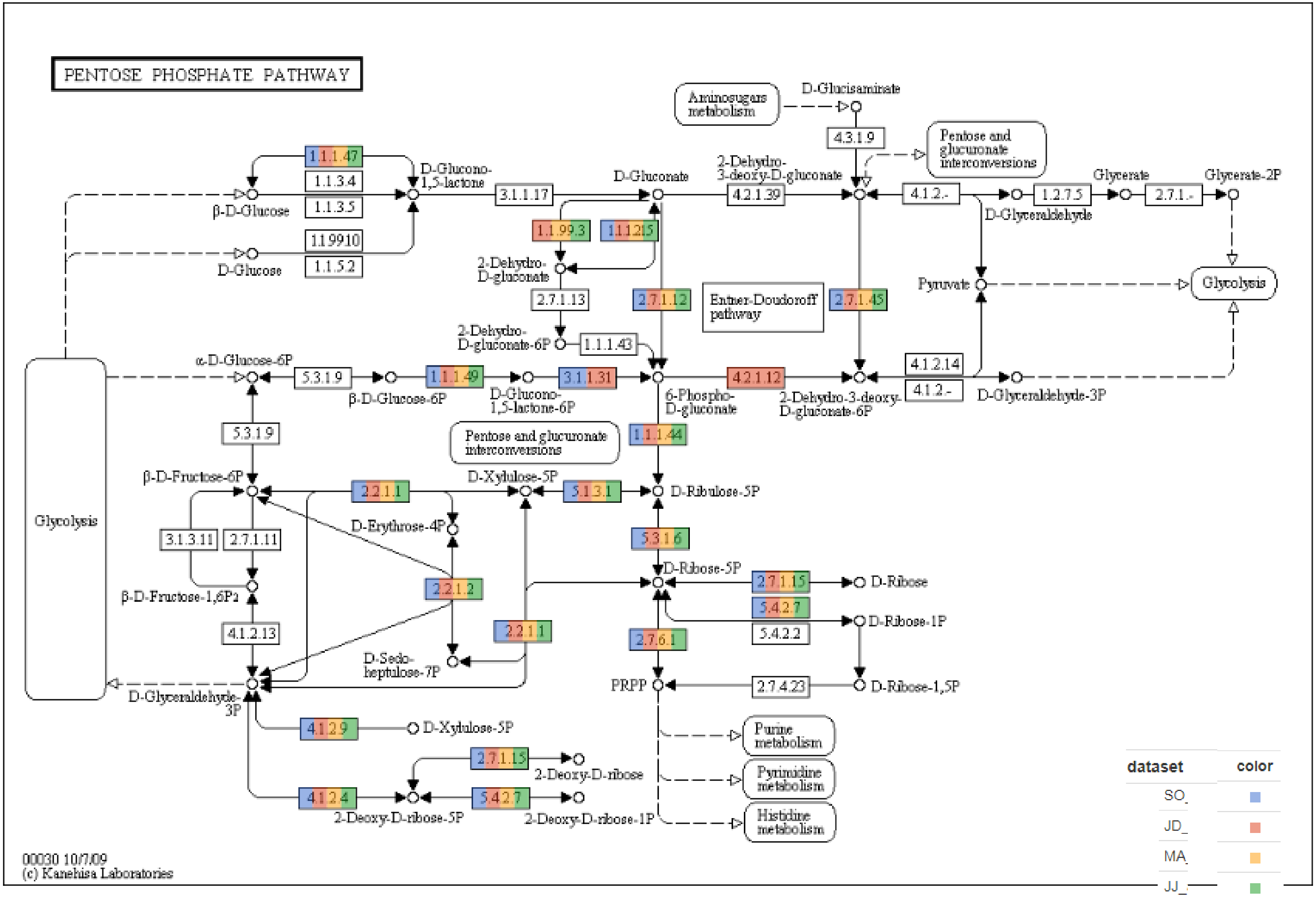

Supplementary Figure S6. KEGG pathway of Pentose phosphate pathway identified from kinema samples. The enzymes of kinema samples (SO, DJ, MA, and JJ) mapped in pathway are represented by different colors.

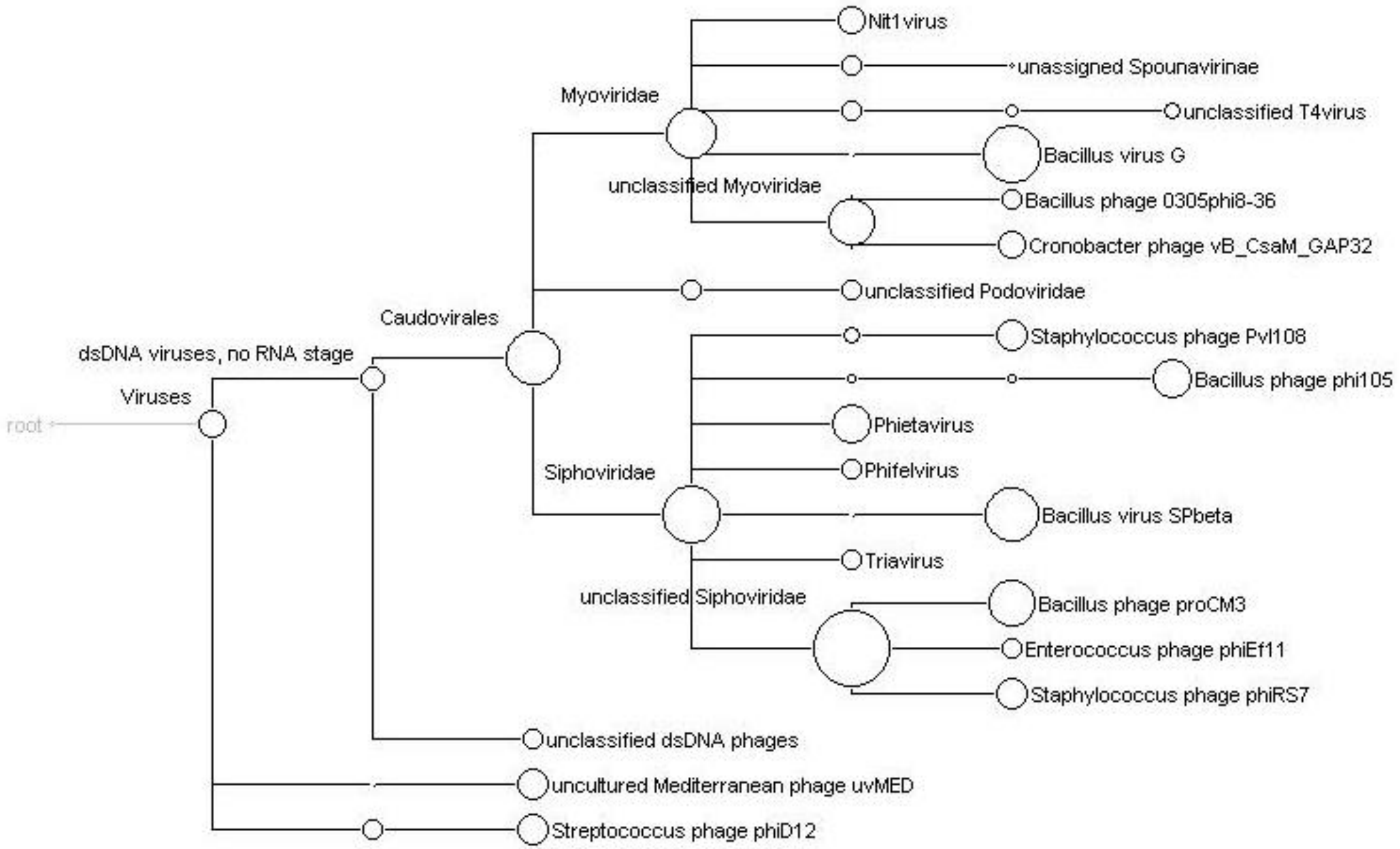

Supplementary Figure S7. Analysis of enriched phage taxonomy in kinema samples using Core Biome. Enriched phages at their relative taxonomic level are represented by large circles.

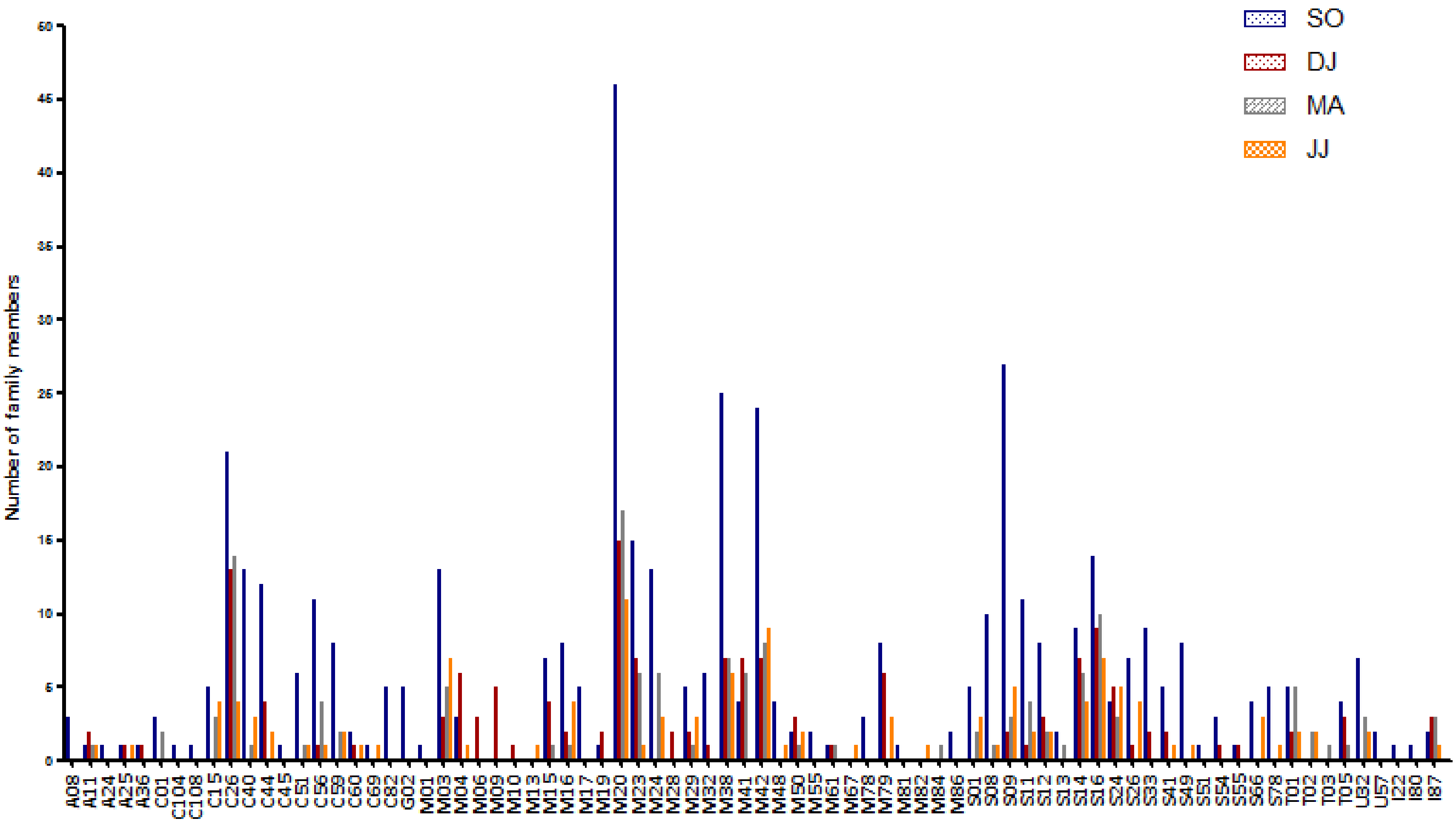

Supplementary Figure S8. Identification and classification of proteases from kinema metagenome SO, DJ, MA, and JJ. Proteases belonging to family of Aspartic (A), Cysteine (C), Glutamic (G), Metallo (M), Serine (S), Threonine (T), Unknown (U), and Inhibitor (I) are represented by their respective MEROPS family.

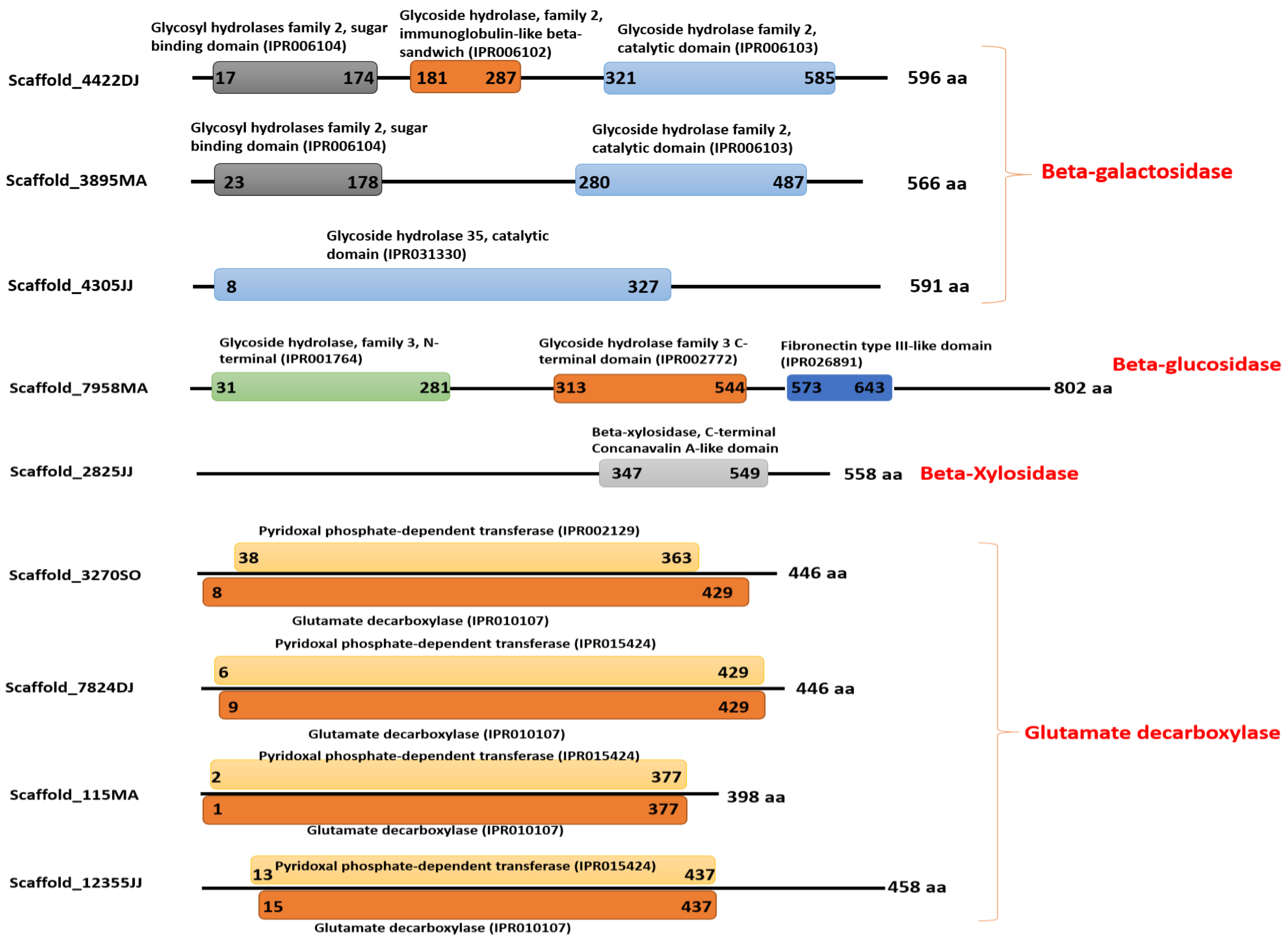

Supplementary Figure S9. Domain architecture of the novel gene involved in carbohydrate transformation and GABA production. The conserved domains in amino-acid sequences of Beta-galactosidase, Beta-glucosidase, Beta-xylosidase, and Glutamate decarboxylase are represented by numbers.

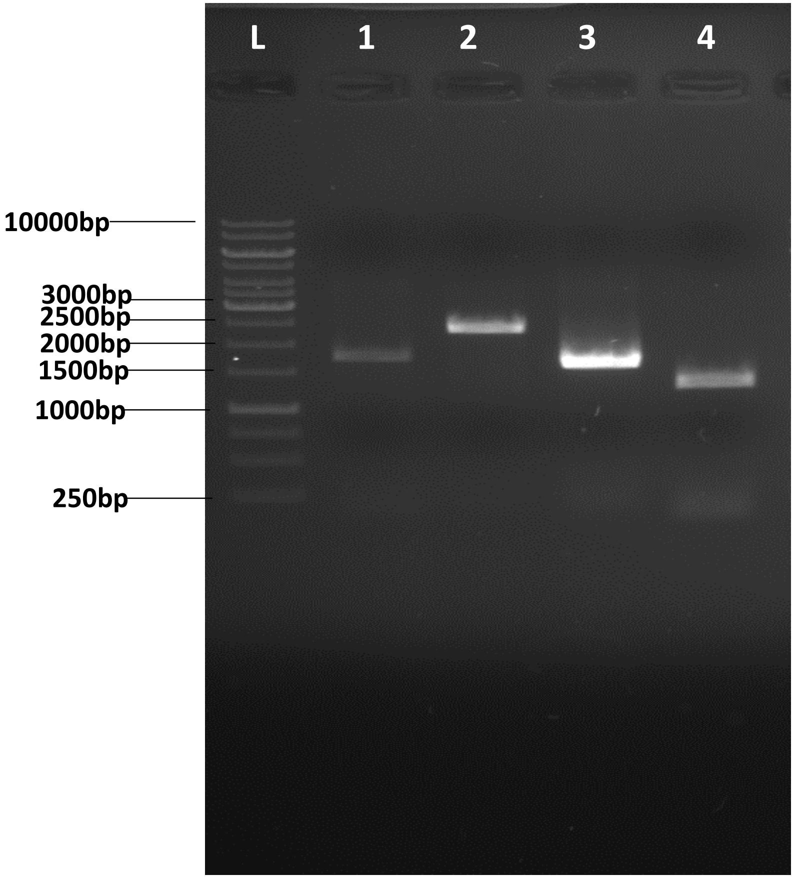

Supplementary Figure S10. Polymerase chain reaction (PCR) amplification of (1) Beta-galactosidase, (2) Beta-glucosidase, (3) Beta-xylosidase, and (4) Glutamate decarboxylase form metagenomics DNA of DJ, MA, JJ, and SO respectively. Amplicon showing the size of 1791bp, 2409bp, 1677bp, and 1341bp are shown in lane 1 to 4 respectively. Lane L represents loading of 1Kb DNA ladder.

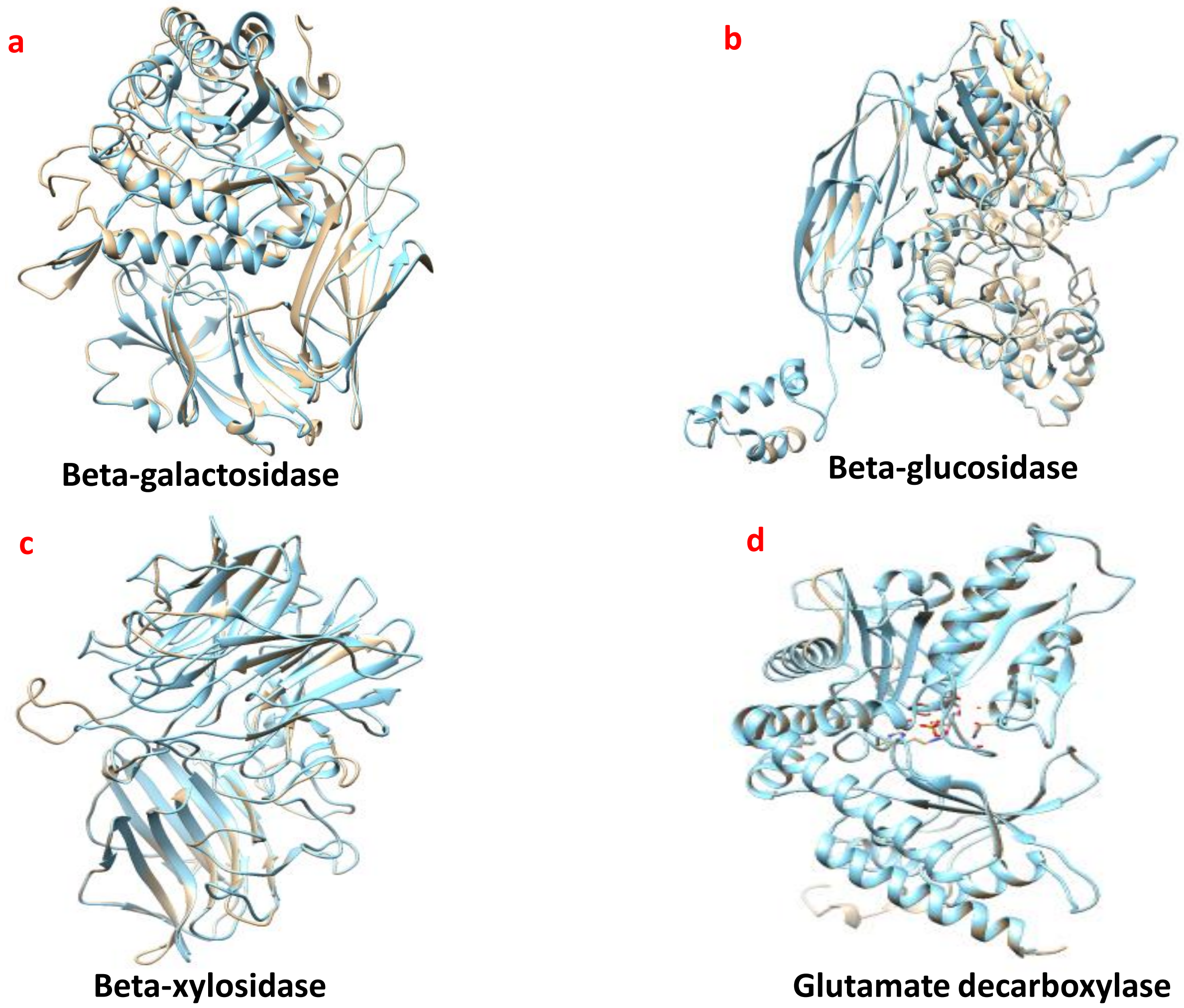

Supplementary Figure S11. Superimposition of putative enzymes on the best-hit template- (a) Beta-galactosidase (template: c3lpgA; Identity%: 22%; RMSD: 0.032), (b) Beta-glucosidase (template: c5wabD; Identity%: 39%; RMSD: 0.192), (c) Beta-xylosidase (template: c1yifC; Identity%: 36%; RMSD: 0.203), and (d) Glutamate decarboxylase (template: c5gp4C; Identity%: 59%; RMSD: 0.059).

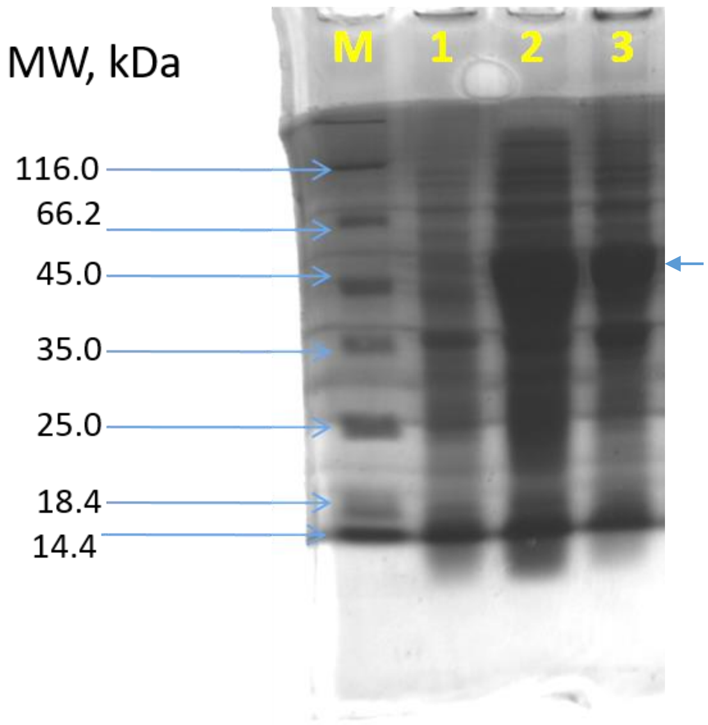

Supplementary Figure S12. SDS-PAGE analysis of crude cell-extract showing glutamate decarboxylase protein expression. Lane M: protein ladder, Lane 1: crude-cells extract of GAD transformed *E. coli* at un-induced state, Lane 2: soluble fraction of crude-cells extract of GAD expressed *E. coli*, and Lane 3: pellet fraction of crude-cells extract of GAD expressed *E. coli*.
